# Response-guided first-line therapy and treatment of relapse in aggressive lymphoma: 10-year follow-up of the PETAL trial

**DOI:** 10.1016/j.bneo.2024.100018

**Published:** 2024-05-21

**Authors:** Ulrich Dührsen, Andreas Bockisch, Bernd Hertenstein, Imke E. Karsten, Frank Kroschinsky, Michael Heuser, Andreas Hochhaus, Heinz-Gert Höffkes, Dirk Behringer, Gabriele Prange-Krex, Mareike Tometten, Martin Grieshammer, Götz U. Grigoleit, Oliver Schmalz, Karin Jordan, Helga Bernhard, Tobias Gaska, Aristoteles Giagounidis, Roland Schroers, Uwe M. Martens, Gerhard Held, Wolfram Klapper, Karl-Heinz Jöckel, Michael Nonnemacher, Andreas Hüttmann, PETAL Trial Investigators, S. Wilop, M. Tometten, A. Korfel, U. Keller, M. de Wit, F. Weissinger, U. Stark, D. Behringer, U. Bückner, H. Nückel, R. Schroers, G. Trenn, B. Hertenstein, H. Bernhard, M. Heike, M.-A. Wörns, F. Kroschinsky, G. Prange-Krex, A. Giagounidis, G. U. Grigoleit, J. Selbach, S. Petrasch, J. S. Balleisen, J. Schütte, A. Giagounidis, A. Dienst, U. Germing, U. Dührsen, U. von Verschuer, P. Reimer, H.-G. Höffkes, V. Runde, C. Spohn, R. Moeller, H. Dürk, D. Kofahl-Krause, M. Heuser, M. Witzens-Harig, C. Müller-Tidow, U. M. Martens, D. Strumberg, P. La Rosée, A. Hochhaus, H. Link, G. Held, M. Kneba, C. Baldus, R. Naumann, J. M. Chemnitz, K. Schulte, C. Limmroth, A. Schwarzer, D. Niederwieser, U. Platzbecker, G. Heil, M. Schwalenberg, M. Grieshammer, C. Beck, M. Stephany, R. Mesters, I. E. Karsten, H. Held, S. Mahlmann, H. Steiniger, T. Gaska, T. Südhoff, C. Kreisel-Büstgens, E. Moorahrend, G. Maschmeyer, K. Jordan, O. Kloke, M. Klein, T. Höhler, M. Grube, W. Herr, D. Hahn, A. Raghavachar, O. Schmalz, W. Fett, M. Sandmann, T. Krohn, W. Brenner, M. Plotkin, C. Franzius, J. Kotzerke, H. Hautzel, A. Bockisch, A. Hertel, F. M. Bengel, U. Haberkorn, M. Freesmeyer, U. Lützen, A. Klein, R. Kluge, R. Larisch, E. Fricke, J. Holzinger, W. Schäfer, M. Weckesser, F. Nyuyki, W. Römer, I. Brink, J. Marienhagen, G. Pöpperl, K.-H. Jöckel, M. Nonnemacher, J. Rekowski, A. Scherag, M. Neuhäuser

**Affiliations:** 1 Klinik für Hämatologie und Stammzelltransplantation, Universitätsklinikum, Essen, Germany; 2 Klinik für Nuklearmedizin, Universitätsklinikum, Essen, Germany; 3 Medizinische Klinik I, Klinikum Bremen Mitte, Bremen, Germany; 4 Medizinische Klinik A, Universitätsklinikum, Münster, Germany; 5 Medizinische Klinik I, Universitätsklinikum Carl Gustav Carus, Dresden, Germany; 6 Klinik für Hämatologie, Hämostaseologie, Onkologie und Stammzelltransplantation, Medizinische Hochschule, Hannover, Germany; 7 Klinik für Innere Medizin II, Universitätsklinikum, Jena, Germany; 8 Tumorklinik, Klinikum, Fulda, Germany; 9 Klinik für Hämatologie, Onkologie und Palliativmedizin, Augusta-Kranken-Anstalt, Bochum, Germany; 10 Onkologische Gemeinschaftspraxis, Dresden, Germany; 11 Klinik für Hämatologie, Onkologie, Hämostaseologie und Stammzelltransplantation, Uniklinik RWTH Aachen, Aachen, Germany; 12 Centrum für Integrierte Onkologie, Aachen, Bonn, Cologne, Düsseldorf, Aachen, Germany; 13 Universitätsklinik für Hämatologie, Onkologie, Gerinnungsstörungen und Palliativmedizin, Johannes Wesling Klinikum, Minden, Germany; 14 Klinik für Hämatologie, Onkologie und Immunologie, Helios Klinikum, Duisburg, Germany; 15 Klinik für Hämatologie, Onkologie, klinische Infektiologie und Palliativmedizin, Helios Universitätsklinikum, Wuppertal, Germany; 16 Klinik für Hämatologie, Onkologie und Palliativmedizin, Klinikum Ernst von Bergmann, Potsdam, Germany; 17 Medizinische Klinik V, Klinikum, Darmstadt, Germany; 18 Klinik für Hämatologie und Onkologie, Brüderkrankenhaus St Josef, Paderborn, Germany; 19 Klinik für Onkologie, Hämatologie und Palliativmedizin, Marienhospital, Düsseldorf, Germany; 20 Medizinische Klinik, Sektion Hämatologie, Onkologie, Stammzell- und Immuntherapie, Universitätsklinikum Knappschaftskrankenhaus, Bochum, Germany; 21 Klinik für Innere Medizin III, SLK-Kliniken, Heilbronn, Germany; 22 Klinik für Innere Medizin 1, Westpfalz-Klinikum, Kaiserslautern, Germany; 23 Institut für Pathologie, Sektion für Hämatopathologie, Universitätsklinikum Schleswig-Holstein, Kiel, Germany; 24 Institut für Medizinische Informatik, Biometrie und Epidemiologie, Universität Duisburg-Essen, Essen, Germany

## Abstract

The PETAL (Positron Emission Tomography–Guided Therapy of Aggressive Non-Hodgkin Lymphomas) trial investigated whether treatment intensification after 2 cycles of cyclophosphamide, doxorubicin, vincristine, and prednisone (CHOP; plus rituximab [R] for CD20^+^ lymphomas) improved survival in aggressive non-Hodgkin lymphoma. A total of 754 patients (87.5%) had a negative and 108 (12.5%) had a positive interim positron emission tomography (iPET) scan. iPET-positive patients were randomly assigned to receive another 6 (R-)CHOP cycles or 6 blocks of a more intensive protocol. Interim PET-negative patients received another 4 cycles of R-CHOP with or without 2 additional doses of R. After a median follow-up of 4 years, treatment intensification had not improved outcome. These results were confirmed after 10.3 years of follow-up. The present analysis also describes the management of relapse that was part of the study. Among 240 relapsing patients, 94 of 133 autologous transplantation–eligible patients (70.7%) and 16 of 107 ineligible patients (15.0%) received the salvage treatments recommended in the study protocol. Adherence to recommendations had no impact on outcome. Best results were seen after allogeneic transplantation, followed by autologous transplantation and treatment without transplantation (5-year overall survival rate, 64.3% vs 45.5% vs 22.6%), but patients undergoing allogeneic transplantation were significantly younger and their disease was well controlled at the time of transplantation. Early outcome prediction by iPET, alone or in combination with other methods, is a powerful tool to investigate the value of immunological treatment options for patients with a poor response to chemotherapy. This trial was registered at ClinicalTrials.gov as #NCT00554164.

## Key Points

• Interim PET predicted outcome in aggressive non-Hodgkin lymphoma, but iPET-based treatment changes failed to improve outcome.
• Treatment of relapse often differed from protocol-based recommendations, with best results after allogeneic transplantation.

## Visual Abstract

**Figure:**
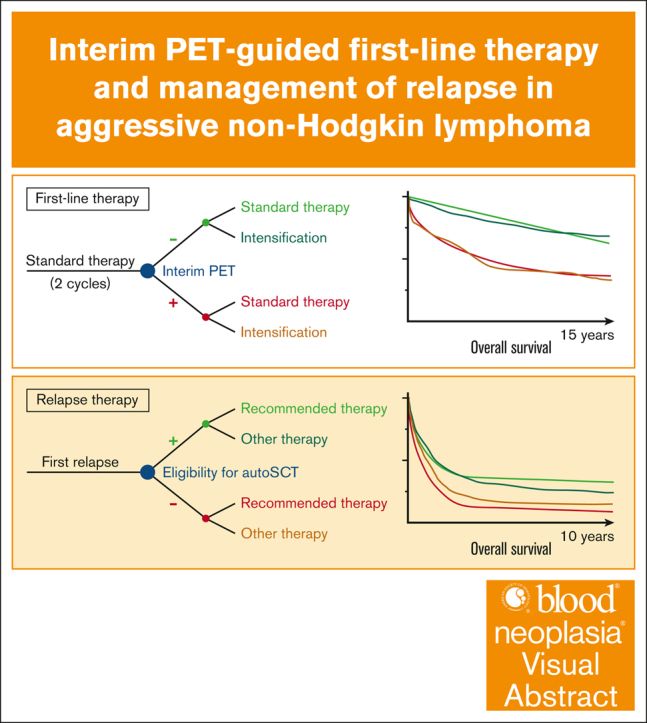

## Introduction

Interim positron emission tomography (iPET) predicts outcome in aggressive lymphomas.^1^^,^^2^ The PETAL (Positron Emission Tomography–Guided Therapy of Aggressive Non-Hodgkin Lymphomas) trial investigated whether treatment intensification after 2 cycles of cyclophosphamide, doxorubicin, vincristine, and prednisone (CHOP; plus rituximab [R] for CD20^+^ lymphomas) improved survival.^3^ After a median follow-up of 4 years, we reported that intensification failed to show a positive impact on outcome.^3^

The study protocol also made recommendations for the treatment of progression or relapse (collectively referred to as “relapse”) and asked for documentation of all lines of salvage therapy. Outcome after relapse was not reported in our original publication because of short follow-up.^3^

Here, we report the final results of the PETAL trial for both first-line and salvage therapy.

## Methods

The PETAL trial was registered at ClinicalTrials.gov as #NCT00554164 and approved by the German Federal Institute for Drugs and Medical Devices and the ethics committees of the participating centers. The study was performed according to the principles of the Declaration of Helsinki. All patients gave written informed consent. The first patient was included in November 2007, recruitment was stopped in December 2012, und the database was locked in July 2023.

Patients aged 18 to 80 years with all types of aggressive non-Hodgkin lymphoma except Burkitt lymphoma and primary central nervous system lymphoma and a positive baseline PET scan were eligible. Interim PET was evaluated using the ΔSUV_max_ method (maximum standardized uptake value [SUV_max_]) that has been shown to be superior to the commonly used Deauville scale for outcome prediction.^1^^,^^4^ An SUV_max_ reduction of ≤66% compared with baseline defined a positive iPET.^5^

After 2 (R-)CHOP cycles, iPET-positive patients were randomly assigned to receive another 6 (R-)CHOP cycles or 6 blocks of an intensive methotrexate-based Burkitt lymphoma protocol.^6^ Interim PET-negative patients with CD20^+^ lymphomas were randomly assigned to receive another 4 cycles of R-CHOP with or without 2 additional doses of R. Randomization of iPET-positive patients was performed during the entire recruitment phase, whereas randomization of iPET-negative patients with CD20^+^ lymphomas was restricted to the period between February 2010 and October 2011, when the recruitment goal was reached. Before February 2010, all such patients received 6 cycles of R-CHOP, whereas, after October 2011, they received 6 cycles of R-CHOP with 2 additional doses of R. Patients with CD20^−^ lymphomas and a negative iPET uniformly received 6 cycles of CHOP. A CONSORT diagram showing recruitment and treatment allocation has been published previously.^3^

Recommended first-relapse treatments were 2 cycles of (R-)DHAP (dexamethasone, high-dose cytarabine, and cisplatin) followed by high-dose BEAM(-R) (carmustine, etoposide, cytarabine, and melphalan) and autologous blood stem cell transplantation (auto-SCT) for auto-SCT–eligible patients,^7^ and 6 cycles of (R-)ESHAP (etoposide, methylprednisolone, high-dose cytarabine, and cisplatin) for auto-SCT–ineligible patients.^8^

The primary end point of first-line randomization was event-free survival, defined as progression, relapse, treatment incompatible with the protocol (including radiotherapy), toxicity-related discontinuation, or death of any cause. It was tested using the log-rank test at a 2-sided significance level of .05. The sample size calculation for the primary end point has been published previously.^3^ Other end points of first-line and relapse therapy included time to progression, progression-free survival, and overall survival (OS). All authors had access to the primary data. The statistical analyses were performed by U.D., K.-H.J., and M.N. using IBM SPSS Statistics, version 29.0 (Armonk, NY).

Further details of treatments, definitions, and statistical methods are provided in supplemental Data, including a synopsis of the PETAL trial protocol.

## Results

### First-line therapy

**Figure 1.**
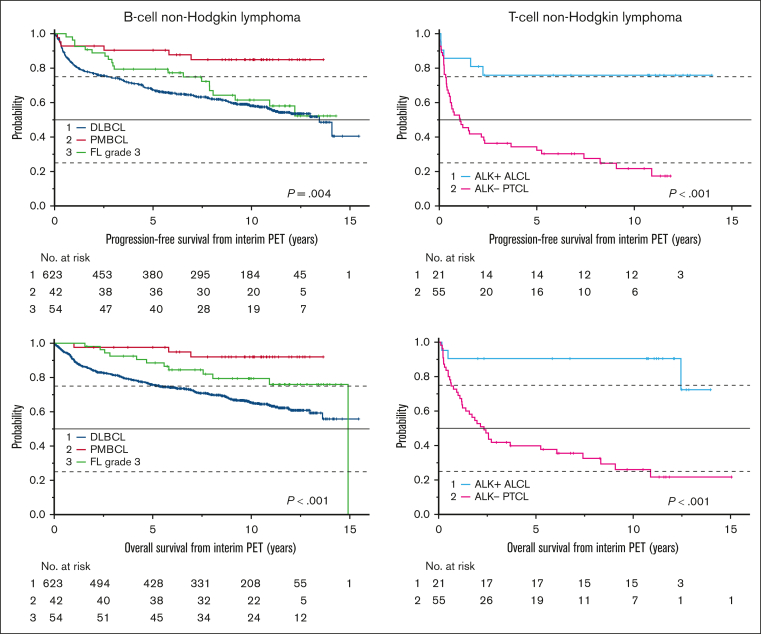
**Outcome by lymphoma subtype.** Progression-free survival (top) and OS (bottom) from the day of iPET for patients with various types of B-cell non-Hodgkin lymphoma (DLBCL, primary mediastinal B-cell lymphoma [PMBCL], follicular lymphoma [FL] grade 3; left) or T-cell non-Hodgkin lymphoma (ALK^+^ ALCL, ALK^−^ peripheral T-cell lymphoma [PTCL]; right). Of 623 patients with DLBCL, 574 had DLBCL alone, and 49 had DLBCL combined with an indolent lymphoma. Of 54 patients with FL grade 3, 25 had FL grade 3a, 17 had FL grade 3b, and 12 had FL grade 3 combined with FL grade 1 or 2. Of 55 patients with ALK^−^ PTCL, 13 had ALK^−^ ALCL, 18 had angioimmunoblastic T-cell lymphoma, 20 had PTCL not otherwise specified, and 4 had unclassified T-cell lymphoma. Because the subgroups within the DLBCL, FL grade 3, and ALK^−^ PTCL cohorts had similar outcomes (supplemental Figure 1), they were combined in this analysis. No., number; *p*, log-rank test.

Follow-up was updated for 621 of 677 patients (91.7%) who were alive at the time of the first analysis. The median follow-up time was 10.3 years (interquartile range, 7.7-11.9). Table 1, Figure 1, and supplemental Figure 1 detail baseline characteristics and outcomes for the entire study population and histologically defined subgroups.

**Table 1.** **Baseline features, major outcomes, and events after first-line therapy in 862 patients participating in the PETAL trial**

	All patients	DLBCL∗	PMBCL	FL grade 3†	Other aNHL‡	ALK^+^ ALCL	ALK- PTCL§	No aNHL‖
Number of patients	862 (100.0)	623 (72.3)	42 (4.9)	54 (6.3)	43 (5.0)	21 (2.4)	55 (6.4)	24 (2.7)
**Baseline features at study inclusion**								
Age, y (range)	60 (18-80)	62 (18-80)	35 (18-80)	58 (29-80)	61 (28-76)	33 (19-66)	65 (26-79)	62 (21-79)
International Prognostic Index risk group¶								
Low	329 (38.3)	228 (36.7)	25 (59.5)	26 (48.2)	9 (20.9)	16 (76.2)	18 (32.7)	7 (31.8)
Low-intermediate	224 (26.1)	163 (26.3)	9 (21.4)	14 (25.9)	10 (23.3)	5 (23.8)	15 (27.3)	8 (36.4)
High-intermediate	180 (21.0)	130 (20.9)	5 (11.9)	10 (18.5)	17 (39.5)	0 (0.0)	15 (27.3)	3 (13.6)
High	125 (14.6)	100 (16.1)	3 (7.2)	4 (7.4)	7 (16.3)	0 (0.0)	7 (12.7)	4 (18.2)
**Major outcomes**								
Survival rate at 5 y after iPET evaluation#								
Progression-free survival	66.9 (63.8-70.0)	67.5 (63.8-71.2)	90.4 (81.4-99.4)	79.4 (68.6-90.2)	67.4 (53.5-81.3)	75.9 (57.5-94.3)	32.3 (19.8-44.8)	53.6 (33.4-73.8)
OS	75.6 (72.7-78.5)	75.8 (72.5-79.1)	97.6 (92.9-102.3)	88.6 (80.0-97.2)	76.7 (64.2-89.2)	90.5 (78.0-103.0)	39.8 (26.9-52.7)	70.0 (51.4-88.6)
Survival rate at 10 y after iPET evaluation#								
Progression-free survival	57.0 (53.5-60.5)	58.1 (54.0-62.2)	84.9 (73.7-96.1)	61.5 (47.0-76.0)	56.3 (40.8-71.8)	75.9 (57.5-94.3)	21.7 (9.5-33.9)	32.5 (11.9-53.1)
OS	66.0 (62.7-69.3)	65.5 (61.4-69.6)	92.0 (83.4-100.6)	79.4 (67.8-91.0)	68.1 (53.6-82.6)	90.5 (78.0-103.0)	26.0 (12.9-39.1)	65.3 (45.7-84.9)
**Events after first-line therapy**								
Progression or relapse	240 (27.8)	158 (25.4)	4 (9.5)	16 (29.6)	14 (32.6)	5 (23.8)	31 (56.4)	12 (50.0)
Time to first relapse, y (range)	0.8 (0.0-14.1)	0.8 (0.0-14.1)	0.3 (0.2-2.5)	3.0 (0.6-12.2)	0.8 (0.2-7.7)	0.2 (0.1-2.2)	0.5 (0.0-8.3)	1.6 (0.3-9.5)
Type of salvage therapy∗∗								
Supportive care alone	16 (6.7)	12 (7.6)	0 (0.0)	1 (6.3)	0 (0.0)	1 (20.0)	1 (3.2)	1 (8.3)
Chemotherapy, immunotherapy, and/or radiotherapy	127 (52.9)	79 (50.0)	1 (25.0)	8 (50.0)	9 (64.3)	1 (20.0)	20 (64.5)	9 (75.0)
Auto-SCT	69 (28.7)	53 (33.5)	0 (0.0)	5 (31.3)	3 (21.4)	1 (20.0)	6 (19.4)	1 (8.3)
Auto-SCT	28 (11.7)	14 (8.9)	3 (75.0)	2 (12.5)	2 (14.3)	2 (40.0)	4 (12.9)	1 (8.3)
Survival rate at 5 y after first relapse††								
Progression-free survival	25.3 (19.6-31.0)	27.6 (20.3-34.9)	≤25.0‡‡ (−17.5 to 67.5)	34.3 (9.4-59.2)	21.4 (−0.2 to 43.0)	20.0 (−15.1 to 55.1)	6.5 (−2.1 to 15.1)	38.1 (9.3-66.9)
OS	33.5 (27.4-39.6)	33.1 (25.5-40.7)	75.0 (32.5-117.5)	48.1 (23.0-73.2)	35.7 (10.6-60.8)	60.0 (17.1-102.9)	9.7 (−0.7 to 20.1)	54.7 (24.5-84.9)
Second primary malignancies and deaths								
Second primary malignancy	100 (11.6)	72 (11.6)	3 (7.1)	6 (11.1)	6 (14.0)	2 (9.5)	7 (12.7)	4 (16.7)
Time to second primary malignancy, y (range)	4.7 (0.3-13.3)	4.9 (0.3-13.3)	9.1 (4.9-9.6)	2.9 (0.4-4.0)	5.0 (3.0-8.3)	3.7 (2.8-4.5)	3.7 (1.5-8.1)	3.0 (2.3-11.3)
Death	286 (33.2)	206 (33.1)	3 (7.1)	12 (22.2)	15 (34.9)	3 (14.3)	39 (70.9)	8 (33.3)

Numbers are given as n (%) unless otherwise noted.

aNHL, aggressive non-Hodgkin lymphoma; FL, follicular lymphoma; PMBCL, primary mediastinal B-cell lymphoma; PTCL, peripheral T-cell lymphoma.

∗ Including 574 DLBCL and 49 DLBCL combined with FL (n = 43), marginal zone lymphoma (n = 5), or lymphoid granulomatosis grade 2 (n = 1). Because DLBCL alone and DLBCL combined with an indolent lymphoma had similar relapse rates, progression-free survival, and OS (supplemental Figure 1), the 2 groups were combined.

† Including 25 FL grade 3a, 17 FL grade 3b, and 12 FL grade 3 combined with grade 1 or 2. Because the 3 groups had similar relapse rates, progression-free survival, and OS (supplemental Figure 1), they were combined.

‡ Including 4 B-cell lymphomas with features intermediate between DLBCL and Burkitt lymphoma, 3 B-cell lymphomas with features intermediate between DLBCL and Hodgkin lymphoma, 2 plasmablastic lymphomas, and 34 unclassified large B-cell lymphomas.

§ Including 13 ALK^−^ ALCL, 18 angioimmunoblastic T-cell lymphomas, 20 PTCL not otherwise specified, and 4 unclassified PTCL. Because the 4 groups had similar relapse rates, progression-free survival, and OS (supplemental Figure 1), they were combined.

‖ Including 10 FL grade 1 or 2, 7 marginal zone lymphomas, 1 unclassified indolent B-cell lymphoma, 2 mantle cell lymphomas, 2 Burkitt lymphomas, 1 Hodgkin lymphoma, and 1 breast cancer.

¶ Percentages refer to patients with documented data only.

# Kaplan-Meier estimate of the percentage of patients surviving after 5 or 10 years, respectively (95% CI). After undergoing first-line immunochemotherapy as specified in the study protocol, 23 patients received consolidating radiotherapy. This was counted as a treatment-failure event in the event-free survival analysis but not in the progression-free survival or OS analyses (Figure 3).

∗∗ The type of salvage therapy was defined on the basis of the entire disease course (up to 7 lines of salvage therapy), including (1) supportive care alone; (2) chemotherapy, immunotherapy, and/or radiotherapy with or without supportive care, but without transplantation; (3) high-dose chemotherapy with auto-SCT with or without chemotherapy, immunotherapy, radiotherapy, or supportive care, but without allogeneic transplantation; and (4) allogeneic transplantation with or without autologous transplantation, chemotherapy, immunotherapy, radiotherapy, or supportive care.

†† Kaplan-Meier estimate of the percentage of patients surviving after 5 years (95% CI).

‡‡ Indicated time point not yet reached, that is, estimate for an earlier time point.

### Interim PET-based outcome prediction and randomized comparisons

**Figure 2.**
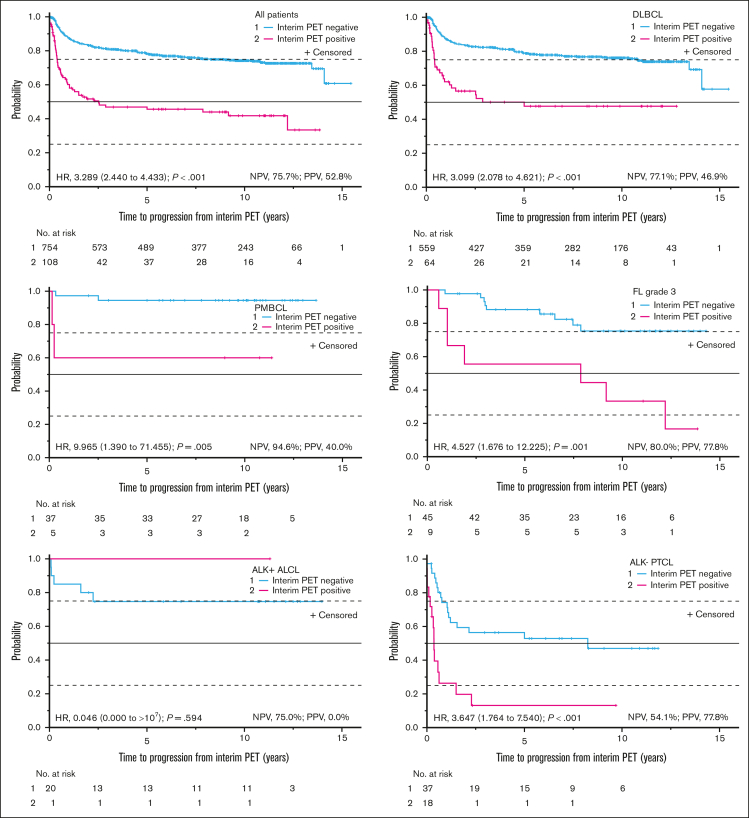
**Outcome by iPET response.** Time to progression from the day of iPET in relation to the iPET result for all patients and for the subgroups of patients with DLBCL, PMBCL, FL grade 3, ALK^+^ ALCL, and ALK^−^ PTCL. The numbers in parentheses represent 95% CIs. The negative predictive values (NPVs) and positive predictive values (PPVs; categorization irrespective of follow-up time) of the iPET result for progression or relapse are also indicated. Similar results were obtained for event-free survival, progression-free survival, and OS (data not shown). No., number; *p*, log-rank test.

**Figure 3.**
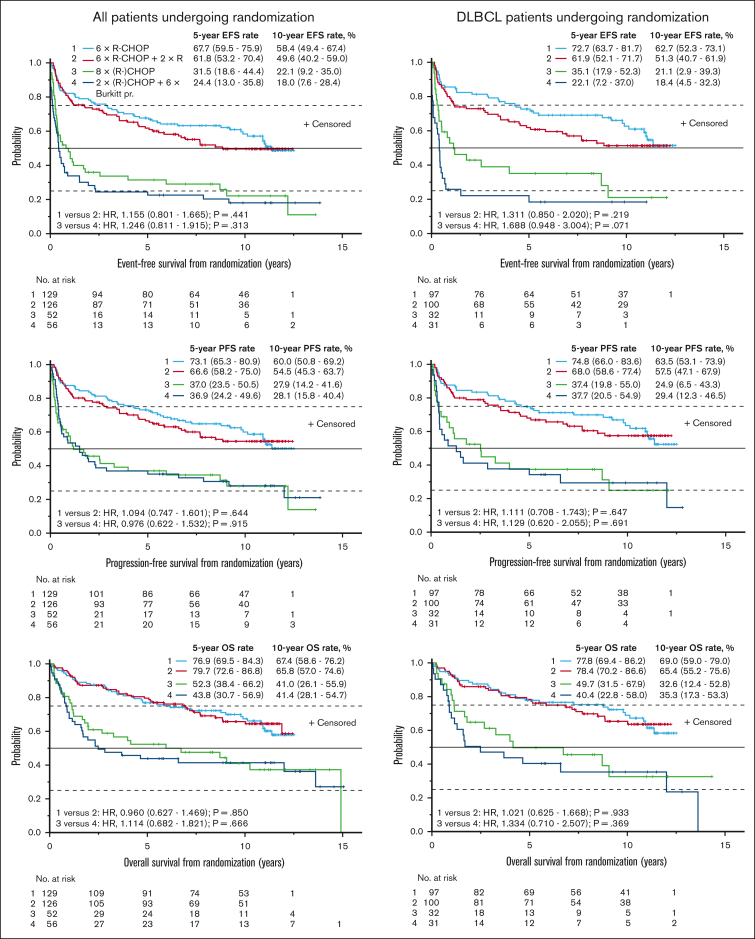
**Outcome by randomly assigned treatment arm.** Event-free survival (EFS, top), progression-free survival (PFS, middle), and OS (bottom) from the day of random assignment for all patients undergoing randomization (left) and for the DLBCL subgroup (right). The top 2 curves in each panel represent iPET-negative patients with CD20^+^ lymphomas, randomly assigned to a total of 6 cycles of R-CHOP with or without 2 additional doses of R. The bottom 2 curves represent iPET-positive patients randomly assigned to a total of 8 cycles of R-CHOP or 2 cycles of R-CHOP followed by 6 blocks of the Burkitt protocol. Baseline stratification factors included age, sex, lymphoma subtype, risk group of the International Prognostic Index, and type of treatment institution.^3^ The numbers in parentheses represent 95% CIs. No., number; *p*, log-rank test.

Interim PET predicted outcome in the entire population and in all subgroups except anaplastic lymphoma kinase (ALK)-positive anaplastic large-cell lymphoma (ALCL; negative predictive value for relapse in the entire population, 75.7%; positive predictive value, 52.8%; and diffuse large B-cell lymphoma [DLBCL] subgroup, negative predictive value of 77.1% and positive predictive value of 46.9%; Figure 2). A total of 754 patients (87.5%) had a negative and 108 (12.5%) had a positive interim scan. Treatment intensification by 2 extra doses of R in the iPET-negative group or a switch from (R-)CHOP to the Burkitt protocol in the iPET-positive group had no statistically significant impact on outcome (Figure 3).

### Progression, relapse, second primary malignancies, and death

**Figure 4.**
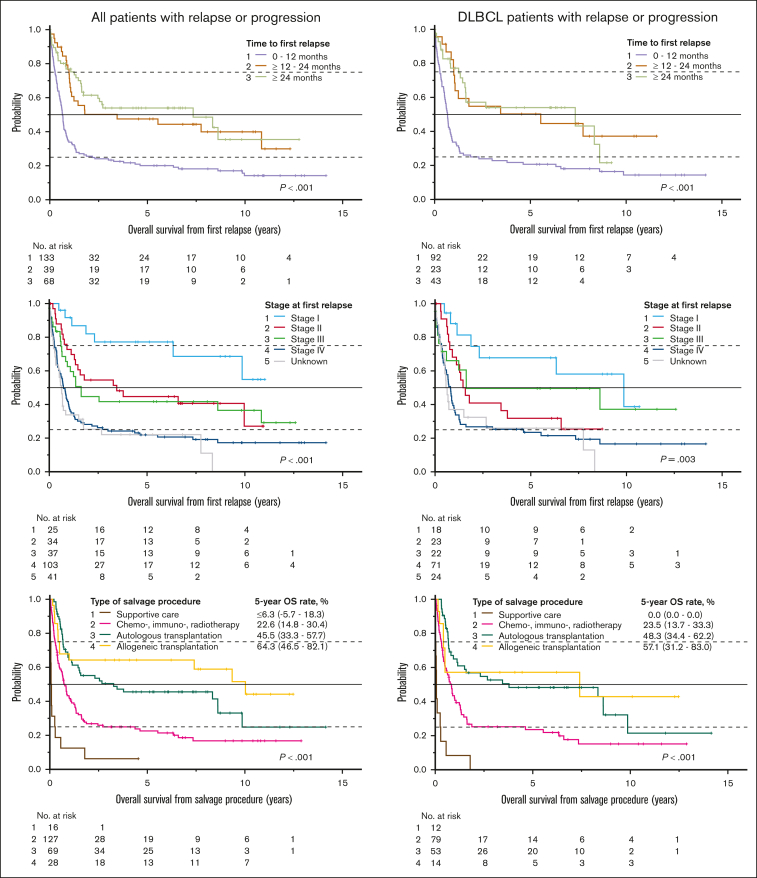
**Outcome after relapse by time to relapse, stage at relapse, and type of salvage therapy.** OS from the day of first relapse for all relapsing patients (left) and for the DLBCL subgroup (right) in relation to the time to first relapse from iPET evaluation (top), the Ann Arbor stage at first relapse (middle), and the type of salvage therapy. The type of salvage therapy was defined on the basis of the entire disease course (up to 7 lines of salvage therapy), including (1) supportive care alone; (2) chemotherapy, immunotherapy, and/or radiotherapy with or without supportive care, but without transplantation; (3) high-dose chemotherapy with auto-SCT with or without chemotherapy, immunotherapy, radiotherapy or supportive care, but without allogeneic transplantation; and (4) allogeneic transplantation with or without autologous transplantation, chemotherapy, immunotherapy, radiotherapy, or supportive care. Chemotherapy/immunotherapy alone, radiotherapy alone, and chemotherapy/immunotherapy consolidated by radiotherapy were combined in 1 group, because outcome after first relapse did not significantly differ among these modalities (supplemental Figure 5). Pairwise comparisons of treatment types were statistically significant (*P* < .001 to *P* = .049), except for autologous vs allogeneic transplantation (*P* = .225 for all relapsing patients, *P* = .791 for the DLBCL subgroup). The numbers in parentheses represent 95% CIs. No., number; *p*, log-rank test.

The median time to first relapse was 0.8 years (range, 0.0-14.1; Table 1). Patients relapsing within the first year had significantly shorter OS from first relapse than patients relapsing later. Patients with stage I relapse fared significantly better than patients in more advanced stages (Figure 4). The lowest postrelapse survival rates were seen in ALK-negative peripheral T-cell lymphoma (Table 1). By July 2023, 286 patients had died. The main causes of death (supplemental Table 1) were lymphoma progression (n = 97, 33.9%), infection (n = 64, 22.4%), cardiovascular events (n = 38, 13.3%), and second primary malignancies (n = 32, 11.2%).

One hundred patients (11.6%) developed a second primary malignancy (1 type, 84; 2 types, 16). The median interval between iPET evaluation and diagnosis of a second primary malignancy was 4.7 years (range, 0.3-13.3). The most frequent diagnoses were prostate (n = 14), colorectal (n = 10), and lung (n = 8) cancer (supplemental Table 2). There was no significant correlation between second cancer incidence and type of lymphoma or type of first-line therapy (data not shown).

### Salvage therapy

Details of salvage therapy were obtained for 238 of 240 (99.2%) relapsing patients (supplemental Table 3). Overall, 224 (93.3%) patients received antineoplastic treatment. The median number of salvage therapy lines was 2 (range, 1-7). There were no statistically significant survival differences between patients receiving 1, 2, or 3 to 7 lines (supplemental Figure 2).

At first relapse, 94 of 133 auto-SCT–eligible patients (70.7%) were started on (R-)DHAP as recommended in the study protocol (median age, 53 years [range, 23-75]; median number of cycles, 2 [range, 1-6]), but only 50 (53.2%) proceeded to BEAM(-R) and auto-SCT. Logistic regression analysis revealed that the only factor to be statistically significantly associated with premature treatment termination was the interval between iPET assessment and relapse. With increasing interval, the risk of not reaching auto-SCT diminished (odds ratio, 0.968 per month; 95% confidence interval [CI], 0.931-0.994; *P* = .019). Numerically, the risk of premature treatment termination appeared higher in females, patients above age 60, patients with T-cell lymphoma, and patients with stage II to IV relapse, but none of these conditions reached statistical significance (supplemental Table 4). At the discretion of the investigators, 30 patients received other high-dose chemotherapy combinations in first relapse, and 7 underwent auto-SCT as second-, third- or 4th-line salvage therapy (4 after failing (R-)DHAP in first line). Sixteen of 107 auto-SCT-ineligible patients (15.0%) were treated with (R-)ESHAP as recommended in the protocol (median age, 72 years [range, 61-79]; median number of cycles, 4 [range, 1-6]), but, due to progression, only ten (62.5%) received more than 2 cycles.

Ineligibility for auto-SCT was associated with inferior survival in the DLBCL subgroup (progression-free survival: hazard ratio [HR], 1.538; 95% CI, 1.069-2.211; *P* = .019; OS: HR, 1.544; 95% CI, 1.059-2.251; *P* = .023; supplemental Figure 3), but not in the total population (supplemental Figure 4). There were no statistically significant survival differences between recommended first-relapse treatments and other types of treatment. This was true for both auto-SCT–eligible and auto-SCT–ineligible patients (supplemental Figures 3 and 4).

Considering all lines of salvage therapy together, 127 patients received chemotherapy, immunotherapy, and/or radiotherapy without transplantation, 69 received auto-SCT without allogeneic SCT (allo-SCT), and 28 received allo-SCT (preceded by auto-SCT in 18 cases). At allo-SCT, 8 patients were in complete remission, 10 were in partial remission, 2 had stable disease, none had progressive disease, and information was missing for 8. The proportion of primary refractory patients, the interval to, and the disease stage at first relapse, were similar among patients receiving chemotherapy, immunotherapy, and/or radiotherapy without transplantation, auto-SCT without allo-SCT, or allo-SCT (Table 2). Age, however, differed significantly (median age for chemotherapy/immunotherapy/radiotherapy vs auto-SCT vs allo-SCT, 68 [range, 23-86] vs 58 [range, 25-76] vs 46 [range, 21-67] years; *P* < .001). OS was best after allo-SCT, although the difference between autologous and allogeneic transplantation did not reach statistical significance (Figure 4). Six patients (21.4%) died of allo-SCT–related complications (fungal pneumonia [2 cases], hepato-intestinal graft-versus-host disease, pleuro-parenchymal fibroelastosis, pancreatitis, and myocardial infarction).

**Table 2.** **Features of relapse and type of salvage therapy in 240 patients participating in the PETAL trial**

	Type of salvage therapy∗				*P*
	Supportive care	Chemotherapy, immunotherapy, and/or radiotherapy	Autologous transplantation	Allogeneic transplantation	
Number of patients	16	127	69	28	
Interval to first relapse, y (range)	0.8 (0.1-8.3)	0.8 (0.0-14.1)	0.9 (0.2-10.4)	0.6 (0.1-6.5)	.508†
Age at first relapse, y (range)	74 (50-82)	68 (23-86)	58 (25-76)	46 (21-67)	<.001†
**Type of treatment failure leading to first relapse**					
Progression on therapy (refractoriness)	3 (18.8%)	30 (23.6%)	15 (21.7%)	6 (21.4%)	.967‡
Relapse after remission	13 (81.2%)	97 (76.4%)	54 (78.3%)	22 (78.6%)	
**Disease stage at first relapse**					
I	1 (6.3%)	12 (9.4%)	12 (17.4%)	0 (0.0%)	.1467‡
II	1 (6.3%)	15 (11.8%)	12 (17.4%)	6 (21.4%)	
III	2 (12.5%)	16 (12.6%)	11 (15.9%)	8 (28.6%)	
IV	8 (50.0%)	61 (48.1%)	23 (33.3%)	11 (39.3%)	
Unknown	4 (25.0%)	23 (18.1%)	11 (15.9%)	3 (10.7%)	

∗ Salvage therapy was defined on the basis of the entire disease course after first relapse, including (1) supportive care alone; (2) chemotherapy, immunotherapy, and/or radiotherapy with or without supportive care, but without transplantation; (3) high-dose chemotherapy with auto-SCT with or without chemotherapy, immunotherapy, radiotherapy, or supportive care, but without allogeneic transplantation; and (4) allogeneic transplantation with or without autologous transplantation, chemotherapy, immunotherapy, radiotherapy, or supportive care. Chemotherapy/immunotherapy alone, radiotherapy alone, and chemotherapy/immunotherapy consolidated by radiotherapy were combined in 1 group, because outcome after first relapse did not significantly differ among these modalities (supplemental Figure 5).

† Kruskal-Wallis test.

‡ χ^2^ test.

**Figure 5.**
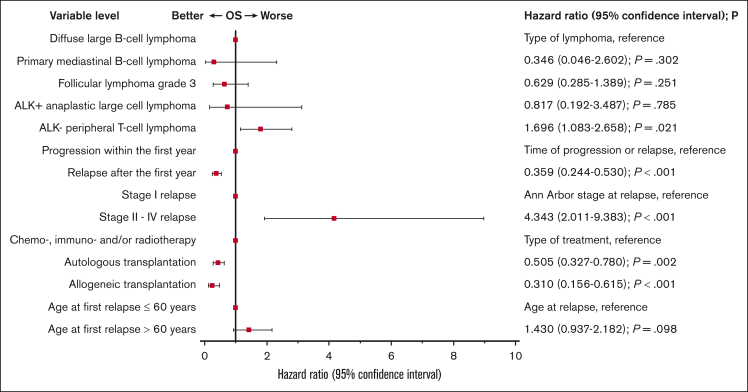
**Forest plot showing multivariable Cox regression analysis of factors determining the OS from the day of first progression or relapse in patients participating in the PETAL trial.** The analysis was restricted to 199 progressing or relapsing patients with a well-defined diagnosis (DLBCL, 146; PMBCL, 4; FL grade 3, 15; ALK^+^ ALCL, 4; ALK^−^ PTCL, 30) fulfilling the study inclusion criteria and receiving antineoplastic treatment for relapse or progression (chemotherapy, immunotherapy, and/or radiotherapy without transplantation, 109; auto-SCT without allogeneic transplantation, 65; and allogeneic transplantation, 25). Overall, 112 patients relapsed within the first year after iPET evaluation, and 87 relapsed later. At the time of first relapse, 21 patients had stage I relapse, 178 had stage II to IV (including 41 patients with unknown stage whose disease course was indistinguishable from that of patients with stage IV disease [Figure 4]), 91 were aged ≤60 years, and 108 were older. Age also failed to reach statistical significance when it was included as a continuous variable into the model (HR, 1.016 per year; 95% CI, 0.999-1.034; *P* = .068). There were 135 events (deaths) among 199 patients.

Multivariable Cox regression analysis revealed lymphoma subtype (bad, ALK-negative peripheral T-cell lymphoma; HR, 1.696; 95% CI 1.083-2.658; *P* = .021), time of relapse (good, >1 year after iPET; HR, 0.359; 95% CI, 0.244-0.530; *P* < .001), stage (bad, stage II-IV; HR, 4.343; 95% CI, 2.011-9.383; *P* < .001), and type of salvage therapy (good, autologous transplantation; HR, 0.505; 95% CI, 0.327-0.780; *P* = .002; good, allogeneic transplantation; HR, 0.310; 95% CI, 0.156-0.615; *P* < .001) to be independently associated with OS. Age failed to reach statistical significance (Figure 5).

## Discussion

In the PETAL trial, iPET predicted 10-year outcome of aggressive non-Hodgkin lymphoma. However, iPET-based treatment intensification did not improve survival. The positive predictive value of iPET was only 50%, that is, half of the iPET-positive patients did well on continued standard therapy and therefore did not need a treatment change. The positive predictive value of the interim assessment may be improved by combining iPET with other markers of treatment response.^9^ Among these, the most promising candidate is circulating tumor DNA whose prognostic value has been shown to be independent of iPET response.^10^ Patients with poor metabolic and molecular responses to first-line therapy may benefit from immunological approaches that have recently been established in the relapsed setting, such as chimeric antigen receptor T cells^11^, ^12^, ^13^ or bispecific antibodies.^14^^,^^15^ Prospective studies are required to assess the value of a switch from chemotherapy to immunotherapy in such patients. The negative predictive value of iPET was close to 80%, supporting the use of iPET alone in treatment de-escalation trials. Two such studies have shown recently that treatment can be shortened in limited-stage DLBCL if the interim scan is negative.^16^^,^^17^

Interim PET was predictive of outcome in all lymphoma entities except ALK-positive ALCL. Failure to predict outcome in this entity may have been related to small patient numbers and a paucity of events in a disease with excellent long-term outcome. However, similar observations were also made in another iPET study.^2^ In contrast to other aggressive non-Hodgkin lymphomas, the prognostic value of iPET in ALK-positive ALCL appears insufficiently defined.

Adherence to salvage therapy recommendations was better for auto-SCT–eligible than for auto-SCT–ineligible patients, reflecting the lack of a therapeutic standard for the latter. Adherence to treatment recommendations had no impact on outcome. The study confirmed the prognostic importance of time^7^ and stage of relapse,^18^ and, for DLBCL, the superiority of auto-SCT over immunochemotherapy alone.^19^ Five-year survival rates were ∼20% for salvage therapy without transplantation, 45% for auto-SCT, and 60% for allo-SCT, which compares favorably with earlier reports on allo-SCT.^20^, ^21^, ^22^, ^23^ However, relapsed patients undergoing allo-SCT were highly selected in the PETAL trial. First, they were significantly younger than patients receiving other treatments. Second, 40% of patients with available information received allo-SCT to consolidate a previously achieved complete remission, half were in partial remission, and none progressed during the previous line of therapy. Patient selection precludes an unbiased comparison of allo-SCT with other treatments for relapsed aggressive lymphoma.

In conclusion, iPET predicted long-term outcome of aggressive non-Hodgkin lymphoma, but iPET-based intensification of chemotherapy failed to improve survival. Whether modern immunological treatment approaches are better suited to achieve this goal should be tested in prospective studies. The management of relapse has changed considerably since the initiation of the PETAL trial. Adherence to protocol-based recommendations had no impact on outcome. Whether this also holds true in an era of novel treatment options, requires further study.

Conflict-of-interest disclosure: The authors declare no competing financial interests.

A complete list of the members of the PETAL (Positron Emission Tomography–Guided Therapy of Aggressive Non-Hodgkin Lymphomas) trial investigators appears in the supplemental Appendix.

## Supplementary Material

Supplemental Methods, Tables, Figures, Appendix, and Study Protocol
