# Dührsen U, Bockisch A, Hertenstein B, et al. Response-guided first-line therapy and treatment of relapse in aggressive lymphoma: 10-year follow-up of the PETAL trial. *Blood Neoplasia*. 2024;1(3):100018.

**DOI:** 10.1016/j.bneo.2026.100192

**Published:** 2026-02-10

Page 3: in Table 1, under “Events after first-line therapy,” the seventh line in the first column should read “Allo-SCT,” not “Auto-SCT.” The corrected Table 1 is shown below:

**Table 1.** **Baseline features, major outcomes, and events after first-line therapy in 862 patients participating in the PETAL trial**

	All patients	DLBCL∗	PMBCL	FL grade 3†	Other aNHL‡	ALK^+^ ALCL	ALK- PTCL§	No aNHL‖
Number of patients	862 (100.0)	623 (72.3)	42 (4.9)	54 (6.3)	43 (5.0)	21 (2.4)	55 (6.4)	24 (2.7)
**Baseline features at study inclusion**								
Age, y (range)	60 (18-80)	62 (18-80)	35 (18-80)	58 (29-80)	61 (28-76)	33 (19-66)	65 (26-79)	62 (21-79)
International Prognostic Index risk group¶								
Low	329 (38.3)	228 (36.7)	25 (59.5)	26 (48.2)	9 (20.9)	16 (76.2)	18 (32.7)	7 (31.8)
Low-intermediate	224 (26.1)	163 (26.3)	9 (21.4)	14 (25.9)	10 (23.3)	5 (23.8)	15 (27.3)	8 (36.4)
High-intermediate	180 (21.0)	130 (20.9)	5 (11.9)	10 (18.5)	17 (39.5)	0 (0.0)	15 (27.3)	3 (13.6)
High	125 (14.6)	100 (16.1)	3 (7.2)	4 (7.4)	7 (16.3)	0 (0.0)	7 (12.7)	4 (18.2)
**Major outcomes**								
Survival rate at 5 y after iPET evaluation#								
Progression-free survival	66.9 (63.8-70.0)	67.5 (63.8-71.2)	90.4 (81.4-99.4)	79.4 (68.6-90.2)	67.4 (53.5-81.3)	75.9 (57.5-94.3)	32.3 (19.8-44.8)	53.6 (33.4-73.8)
OS	75.6 (72.7-78.5)	75.8 (72.5-79.1)	97.6 (92.9-102.3)	88.6 (80.0-97.2)	76.7 (64.2-89.2)	90.5 (78.0-103.0)	39.8 (26.9-52.7)	70.0 (51.4-88.6)
Survival rate at 10 y after iPET evaluation#								
Progression-free survival	57.0 (53.5-60.5)	58.1 (54.0-62.2)	84.9 (73.7-96.1)	61.5 (47.0-76.0)	56.3 (40.8-71.8)	75.9 (57.5-94.3)	21.7 (9.5-33.9)	32.5 (11.9-53.1)
OS	66.0 (62.7-69.3)	65.5 (61.4-69.6)	92.0 (83.4-100.6)	79.4 (67.8-91.0)	68.1 (53.6-82.6)	90.5 (78.0-103.0)	26.0 (12.9-39.1)	65.3 (45.7-84.9)
**Events after first-line therapy**								
Progression or relapse	240 (27.8)	158 (25.4)	4 (9.5)	16 (29.6)	14 (32.6)	5 (23.8)	31 (56.4)	12 (50.0)
Time to first relapse, y (range)	0.8 (0.0-14.1)	0.8 (0.0-14.1)	0.3 (0.2-2.5)	3.0 (0.6-12.2)	0.8 (0.2-7.7)	0.2 (0.1-2.2)	0.5 (0.0-8.3)	1.6 (0.3-9.5)
Type of salvage therapy∗∗								
Supportive care alone	16 (6.7)	12 (7.6)	0 (0.0)	1 (6.3)	0 (0.0)	1 (20.0)	1 (3.2)	1 (8.3)
Chemotherapy, immunotherapy, and/or radiotherapy	127 (52.9)	79 (50.0)	1 (25.0)	8 (50.0)	9 (64.3)	1 (20.0)	20 (64.5)	9 (75.0)
Auto-SCT	69 (28.7)	53 (33.5)	0 (0.0)	5 (31.3)	3 (21.4)	1 (20.0)	6 (19.4)	1 (8.3)
Allo-SCT	28 (11.7)	14 (8.9)	3 (75.0)	2 (12.5)	2 (14.3)	2 (40.0)	4 (12.9)	1 (8.3)
Survival rate at 5 y after first relapse††								
Progression-free survival	25.3 (19.6-31.0)	27.6 (20.3-34.9)	≤25.0‡‡ (−17.5 to 67.5)	34.3 (9.4-59.2)	21.4 (−0.2 to 43.0)	20.0 (−15.1 to 55.1)	6.5 (−2.1 to 15.1)	38.1 (9.3-66.9)
OS	33.5 (27.4-39.6)	33.1 (25.5-40.7)	75.0 (32.5-117.5)	48.1 (23.0-73.2)	35.7 (10.6-60.8)	60.0 (17.1-102.9)	9.7 (−0.7 to 20.1)	54.7 (24.5-84.9)
Second primary malignancies and deaths								
Second primary malignancy	100 (11.6)	72 (11.6)	3 (7.1)	6 (11.1)	6 (14.0)	2 (9.5)	7 (12.7)	4 (16.7)
Time to second primary malignancy, y (range)	4.7 (0.3-13.3)	4.9 (0.3-13.3)	9.1 (4.9-9.6)	2.9 (0.4-4.0)	5.0 (3.0-8.3)	3.7 (2.8-4.5)	3.7 (1.5-8.1)	3.0 (2.3-11.3)
Death	286 (33.2)	206 (33.1)	3 (7.1)	12 (22.2)	15 (34.9)	3 (14.3)	39 (70.9)	8 (33.3)

Numbers are given as n (%) unless otherwise noted.

aNHL, aggressive non-Hodgkin lymphoma; FL, follicular lymphoma; PMBCL, primary mediastinal B-cell lymphoma; PTCL, peripheral T-cell lymphoma.

∗ Including 574 DLBCL and 49 DLBCL combined with FL (n = 43), marginal zone lymphoma (n = 5), or lymphoid granulomatosis grade 2 (n = 1). Because DLBCL alone and DLBCL combined with an indolent lymphoma had similar relapse rates, progression-free survival, and OS (supplemental Figure 1), the 2 groups were combined.

† Including 25 FL grade 3a, 17 FL grade 3b, and 12 FL grade 3 combined with grade 1 or 2. Because the 3 groups had similar relapse rates, progression-free survival, and OS (supplemental Figure 1), they were combined.

‡ Including 4 B-cell lymphomas with features intermediate between DLBCL and Burkitt lymphoma, 3 B-cell lymphomas with features intermediate between DLBCL and Hodgkin lymphoma, 2 plasmablastic lymphomas, and 34 unclassified large B-cell lymphomas.

§ Including 13 ALK^−^ ALCL, 18 angioimmunoblastic T-cell lymphomas, 20 PTCL not otherwise specified, and 4 unclassified PTCL. Because the 4 groups had similar relapse rates, progression-free survival, and OS (supplemental Figure 1), they were combined.

‖ Including 10 FL grade 1 or 2, 7 marginal zone lymphomas, 1 unclassified indolent B-cell lymphoma, 2 mantle cell lymphomas, 2 Burkitt lymphomas, 1 Hodgkin lymphoma, and 1 breast cancer.

¶ Percentages refer to patients with documented data only.

# Kaplan-Meier estimate of the percentage of patients surviving after 5 or 10 years, respectively (95% CI). After undergoing first-line immunochemotherapy as specified in the study protocol, 23 patients received consolidating radiotherapy. This was counted as a treatment-failure event in the event-free survival analysis but not in the progression-free survival or OS analyses (Figure 3).

∗∗ The type of salvage therapy was defined on the basis of the entire disease course (up to 7 lines of salvage therapy), including (1) supportive care alone; (2) chemotherapy, immunotherapy, and/or radiotherapy with or without supportive care, but without transplantation; (3) high-dose chemotherapy with auto-SCT with or without chemotherapy, immunotherapy, radiotherapy, or supportive care, but without allogeneic transplantation; and (4) allogeneic transplantation with or without autologous transplantation, chemotherapy, immunotherapy, radiotherapy, or supportive care.

†† Kaplan-Meier estimate of the percentage of patients surviving after 5 years (95% CI).

‡‡ Indicated time point not yet reached, that is, estimate for an earlier time point.

