# Supplementary material for: Response-guided first-line therapy and treatment of relapse in aggressive lymphoma: 10-year follow-up of the PETAL trial
Source: Blood Neoplasia. 2024 May 21;1(3):100018. doi: 10.1016/j.bneo.2024.100018 (PMC12082160; doi:10.1016/j.bneo.2024.100018)
Supplement: Supplemental Methods, Tables, Figures, Appendix, and Study Protocol [file BNEO_NEO-2024-000241-mmc1.pdf]

Supplemental data

Response-guided first-line therapy and treatment of relapse in aggressive lymphoma: 10-year follow-up of the PETAL trial

1. Supplemental Methods

Trial design and first-line treatment

Recommended treatments for first relapse

Definition of lines of salvage therapy

Statistical analysis

2. Supplemental Tables

Supplemental Table S1 – Cause of death

Supplemental Table S2 – Second primary malignancies

Supplemental Table S3 – Treatment of relapse or progression

Supplemental Table S4 – Factors influencing completion of high-dose chemotherapy

3. Supplemental Figures

Supplemental Figure S1 – Outcome by DLBCL, FL grade 3 and PTCL subgroup

Supplemental Figure S2 – Outcome by number of lines of salvage therapy

Supplemental Figure S3 – Outcome by choice of salvage therapy in the DLBCL subgroup

Supplemental Figure S4 – Outcome by choice of salvage therapy in the total population

Supplemental Figure S5 – Outcome by non-transplant salvage therapy for first relapse

4. Full list of sites and investigators

5. English synopsis of the most recent version of the protocol

1. Supplemental Methods

Trial design and first-line treatment

Trial design, patient recruitment, details of first-line treatment, and outcome after a median follow-up of 4 years have been described before (*J Clin Oncol*. 2018;36(20):2024-2034).

Recommended treatments for first relapse

The trial protocol recommended specific salvage therapies for patients below and above the age of 60 years. Since the protocol did not specify whether the age limit pertained to the age at study inclusion or the age at relapse and personal experience and published reports (e.g., *J Clin Oncol*. 1999;17(12):3776-3785; *Ann Oncol*. 2003:14(1):140-151) indicated that high-dose chemotherapy with autologous blood stem cell transplantation can be safely administered to fit patients above age 60, while unfit patients below age 60 might not be candidates for the procedure, the recommendations were used for patients deemed eligible or ineligible for transplantation irrespective of age. Eligibility for autologous blood stem cell transplantation was defined by the treating physicians without involvement of the trial office.

a. Patients eligible for high-dose therapy with autologous blood stem cell transplantation

Two cycles of (R-)DHAP (3-week interval)

Rituximab	375 mg/m² iv	day 1
Dexamethasone	40 mg po	days 2 to 5
Cytarabine	2 x 2.000 mg/m² 3h CI	day 3
Cisplatin	100 mg/m² 24h CI	day 2
G-CSF	5 µg/kg daily sc<br>or<br>6 mg single-shot sc	starting on day 4 until recovery of neutrophils or<br>depot preparation given on day 4

In patients with a positive interim PET scan, peripheral blood stem cells were collected immediately after the first treatment cycle following the interim PET evaluation (i.e., after either (R-)CHOP or block A of the Burkitt protocol) and stored until needed. In patients relapsing despite a negative interim scan, the stem cells were harvested after the second cycle of (R-) DHAP. Rituximab was restricted to patients with CD20-positive lymphomas. Computed tomography-based restaging was performed after the second cycle. In case of complete or partial remission, the patients proceeded to high-dose chemotherapy with BEAM(-R). CI, continuous infusion; h, hour; iv, intravenous; po, peroral; sc, subcutaneous.

One cycle of BEAM(-R) followed by autologous blood stem cell transplantation

Carmustine	300 mg/m² iv	day -6
Etoposide	200 mg/m² iv	days -6 to -3
Cytarabine	2 x 200 mg/m² iv	days -6 to -3
Melphalan	140 mg/m² iv	day -2
Blood stem cells	≥ 2 x 10^6 CD34+ cells/kg iv	day 0
Rituximab	375 mg/m² iv	days +1 and +8

Rituximab was restricted to CD20-positive lymphomas, but adherence to the recommendation to administer rituximab in the setting of high-dose chemotherapy was low even for CD20-positive lymphomas. iv, intravenous.

b. Patients ineligible for high-dose therapy with autologous blood stem cell transplantation

Up to 6 cycles of (R-)ESHAP (3-week intervals)

Rituximab	375 mg/m² iv	day 1
Etoposide	40 mg/m² iv	days 2 to 5
Methylprednisolone	500 mg iv	days 2 to 6
Cytarabine	2.000 mg/m² 3h CI	day 6
Cisplatin	25 mg/m² 24h CI	days 2 to 5
G-CSF	5 µg/kg daily sc<br>or<br>6 mg single-shot sc	starting on day 7 until recovery of neutrophils or<br>depot preparation given on day 7

Rituximab was restricted to patients with CD20-positive lymphomas. Computed tomography-based restaging was performed after the third cycle. In case of complete or partial remission, the patients received another 3 cycles of (R-)ESHAP. CI, continuous infusion; h, hour; iv, intravenous; sc, subcutaneous.

Definition of lines of salvage therapy

A new line of salvage therapy was defined as a change in treatment protocol, excluding cases where treatment was changed because of intolerance (e.g., a switch from R-DHAP to another autologous transplantation induction protocol because of nephrotoxicity). High-dose therapy with autologous blood stem cell transplantation was rated as a single line of therapy, including one or more cycles of conventionally dosed induction, one course of high-dose therapy, and autologous transplantation. However, a switch in the induction protocol because of non-response was rated as two separate lines of therapy. Allogeneic transplantation (including the conditioning regimen and possible donor lymphocyte infusions) was rated as a single line of therapy, but the therapy preceding allogeneic transplantation was considered a separate line.

Statistical analysis

The primary endpoint of first-line treatment was event-free survival (EFS), defined as progression, relapse, treatment incompatible with the study protocol (including radiotherapy), toxicity-related discontinuation, and death of any cause. Other time-dependent endpoints for first-line and salvage therapies included time to progression (TTP), defined as progression or relapse, progression-free survival (PFS), defined as progression, relapse, or death of any cause, and overall survival (OS), defined as death of any cause. All analyses of secondary end-points and subgroups were exploratory, applying a two-sided alpha of 0.05. Time-to-event end-points were analyzed using the Kaplan-Meier estimator and the log-rank test. Hazard ratios and the impact of covariates were estimated using Cox proportional hazards regression. Frequencies were compared using the chi^2 test, and continuous variables were compared using the Kruskal-Wallis test. Logistic regression was used to assess the impact of factors on the risk of premature treatment termination in first relapse. The median follow-up time was estimated by the reverse Kaplan-Meier method.

2. Supplemental Tables

Supplemental Table S1

Cause of death in 286 patients participating in the PETAL trial.

Cause of death	Number of patients (%)
Lymphoma progression	97 (33.9)
Infection[a]	64 (22.4)
Cardiovascular disease[b]	38 (13.3)
Second primary malignancy	32 (11.2)
Surgery	4 (1.4)
Respiratory failure	2 (0.7)
Hepatic failure	2 (0.7)
Renal failure	1 (0.3)
Gastrointestinal disease	1 (0.3)
Pancreatitis	1 (0.3)
Radiation encephalopathy	1 (0.3)
Graft-versus-host disease	1 (0.3)
Suicide	1 (0.3)
Old age	1 (0.3)
Unknown cause	40 (14.0)

[a] Including 40 infections temporally (within 6 weeks of therapy) related to treatment of lymphoma (n=38) or a second primary malignancy (n=2), 24 infections unrelated to cancer treatment.

[b] Including 12 cases of heart failure, 8 myocardial infarction, 4 coronary artery disease, 1 aortic aneurysm rupture, 1 ventricular fibrillation, 2 pulmonary embolism, 6 circulatory failure of unknown origin, 4 stroke.

Supplemental Table S2

Second primary malignancies in 100 patients participating in the PETAL trial.

Type of malignancy	Number of patients (%)[a]	Interval from interim PET evaluation (years)
Skin cancer		
Basal cell carcinoma	7 (0.81)	5.3 (4.0-10.8)
Melanoma	4 (0.46)	6.1 (2.4-10.8)
Atypical fibroxanthoma	1 (0.12)	7.3
Head-and-neck cancer		
Oral cavity	4 (0.46)	10.5 (3.2-14.7)
Larynx	1 (0.12)	4.9
Parotid gland	2 (0.23)	6.0 (3.0-9.1)
Gastrointestinal cancer		
Esophagus	1 (0.12)	3.6
Stomach	2 (0.23)	2.8 (2.7-2.9)
Colorectal	10 (1.16)	4.7 (0.3-13.3)
Anal	1 (0.12)	1.0
Liver	1 (0.12)	8.1
Biliary tract	1 (0.12)	11.2
Pancreas	4 (0.46)	8.1 (1.7-12.1)
Respiratory tract cancer		
Lung	8 (0.93)	2.0 (0.3-9.0)
Urological cancer		
Kidney	1 (0.12)	4.8
Urinary tract	6 (0.70)	5.0 (0.4-10.7)
Gynecological cancer		
Breast	7 (1.87)[b]	5.8 (2.9-9.0)
Endometrium	1 (0.27)[b]	5.0
Vulva	2 (0.53)[b]	1.7 (0.6-2.9)
Andrological cancer		
Prostate	14 (2.87)[c]	5.2 (1.5-9.6)
Penis	1 (0.21)[c]	1.8
Cerebrospinal cancer		
Meningioma	1 (0.12)	9.0
Glioblastoma	1 (0.12)	2.2

Endocrine cancer		
Thyroid	2 (0.23)	8.3 (7.2-9.5)
Neuroendocrine	1 (0.12)	10.4
Hematopoietic cancer		
Myelodysplastic syndrome	2 (0.23)	6.5 (1.7-11.3)
Acute myeloid leukemia	6 (0.70)	3.0 (1.6-4.1)
Acute lymphoblastic leukemia	1 (0.12)	8.2
Lymphoblastic lymphoma	1 (0.12)	0.7
Idiopathic myelofibrosis	1 (0.12)	9.0
Indolent non-Hodgkin lymphoma	8 (0.93)	6.6 (2.1-12.4)
Aggressive non-Hodgkin lymphoma[d]	4 (0.46)	3.0 (1.4-5.1)
EBV-associated lymphoproliferation	1 (0.12)	2.0
Lymphomatoid granulomatosis	1 (0.12)	0.8
Plasmacytoma	1 (0.12)	0.7
Sarcoma		
Pleomorphic leiomyosarcoma	1 (0.12)	0.3
Cancer of unknown primary	4 (0.46)	7.1 (3.8-12.6)

Abbreviations: EBV, Epstein-Barr virus; PET, positron emission tomography.

[a] Percent of total number of patients participating in the trial (n=862) unless otherwise noted.

[b] Percent of female patients (n=375).

[c] Percent of male patients (n=487).

[d] Two cases of peripheral T-cell lymphoma (PTCL) in patients with diffuse large B-cell lymphoma (DLBCL) and two cases of DLBCL in patients with PTCL.

Supplemental Table S3

Treatment of relapse or progression in 240 patients participating in the PETAL trial.

	**Line of salvage therapy** (number of patients)							
**Type of salvage therapy**	**1**	**2**	**3**	**4**	**5**	**6**	**7**	**All**
Number of patients progressing or relapsing	240	124	66	30	12	5	1	478
Supportive care alone	17[a]	6	2	1	-	-	-	26
Palliative chemo- and /or immunotherapy								
Anthracyclines, anthracenediones[b]	-	2	2	-	2	1	-	7
Bleomycin	-	1	-	1	1	-	-	3
Alkylating agents[c]	-	-	1	2	-	-	-	3
Low-dose methotrexate-based combinations[d]	-	-	1	1	-	-	-	2
Vinca alkaloids[e]	-	-	-	2	1	-	-	3
Others[f]	-	2	1	-	-	-	-	3
Potentially curative chemo- and/or immunotherapy								
Conventionally dosed therapy (not followed by autologous blood stem cell transplantation)								
ESHAP (protocol-based recommendation)	16	-	-	-	-	-	-	16
DHAP (protocol-based recommendation)	44	7	1	1	-	1	-	54
ICE	8	6	-	2	-	-	-	16
Other platinum-based regimens[g]	1	1	-	-	-	-	-	2
DexaBEAM	1	5	-	-	-	-	-	6
Bendamustine[h]	23	12	4	5	1	-	-	45

CHOP-based regimens[i]	7	1	1	-	1	-	-	10
IMVp16	3	2	-	-	-	-	-	5
Other methotrexate-based regimens[j]	18	10	6	-	-	-	-	34
Gemcitabine-based regimens[k]	5	18	10	1	-	-	-	34
Cytarabine-based regimens[l]	1	1	-	-	-	-	-	2
Cladribine-based regimens[m]	-	2	1	-	-	-	-	3
Lenalidomide-based regimens[n]	2	3	5	3	3	1	-	17
Targeted therapy[o]	1	3	3	2	-	-	-	9
Autologous blood stem cell transplantation								
DHAP plus BEAM (protocol-based recommendation)	50	-	-	-	-	-	-	50
ICE plus BEAM	12	-	-	-	-	-	-	12
Other regimens[p]	18	6	1	1	-	-	-	26
Allogeneic blood stem cell transplantation[q]	-	11	12	3	2	-	-	28
Tisagen lecleucel (chimeric antigen receptor T-cells)	-	-	-	-	-	-	1	1
Blinatumumab (bispecific T-cell engager)	-	2	-	-	-	-	-	2
Checkpoint inhibitors[r]	-	2	-	-	-	-	-	2
Radiotherapy alone[s]	11	20	15	3	1	1	-	51
Ibritumumab tiuxetan (radioimmunotherapy)	-	-	-	2	-	-	-	2
Unknown antineoplastic treatment	2	1	-	-	-	1	-	4

NOTE: Chemotherapy was often combined with the anti-CD20 antibody rituximab (rarely obinutuzumab) in patients with CD20-positive lymphomas. Chemotherapy was consolidated by radiotherapy in 28 treatment episodes (salvage line 1, 14; line 2, 9, line 3, 4, line 4, 1).

Abbreviations: BEAM, carmustine/etoposide/cytarabine/melphalan; CHOP, cyclophosphamide/doxorubicin/vincristine/prednisone; DexaBEAM, dexamethasone/carmustine/etoposide/cytarabine/melphalan; DHAP, dexamethasone/high-dose cytarabine/cisplatin; ESHAP, etoposide/ methylprednisolone/high-dose cytarabine/cisplatin; ICE, ifosfamide, carboplatin, etoposide; IMVp19, ifosfamide, methotrexate, etoposide.

[a] At first relapse, one patient with follicular lymphoma grade 3a did not receive treatment other than excisional biopsy of limited-stage disease. At second relapse, she received immunochemotherapy.

[b] Pegylated liposomal doxorubicin, 2; pixantrone, 5.

[c] Cyclophosphamide/prednisone, 1; trofosfamide, 2.

[d] Methotrexate/mercaptopurine, 1; methotrexate/bexarotene/extracorporeal photopheresis, 1.

[e] Vincristine alone, 1; vincristine/cyclophosphamide, 1; vindesine, 1.

[f] Etoposide, 1; fludarabine, 1; bortezomib, 1.

[g] Gemcitabine/dexamethasone/cisplatin (GDP), 1; cisplatin/pemetrexed, 1.

[h] In two cases combined with polatuzumab vedotin.

[i] CHOP, 6; miniCHOP, 2; cyclophosphamide/etoposide/vincristine/prednisone (CEOP), 1; trofosfamide/etoposide/procarbazine/idarubicin/prednisone (TEPIP), 1.

[j] Methotrexate/cytarabine, 1; cyclophosphamide/vincristine/methotrexate/leucovorin/cytarabine (COMLA), 1; German Burkitt's lymphoma protocol (also used for randomization of interim PET positive patients), 18; complex protocols for primary central nervous system lymphoma, 14.

[k] Gemcitabine, 4; gemcitabine/oxaliplatin (GemOx), 24; gemcitabine/cisplatin, 1; gemcitabine/vinorelbine, 2; ifosfamide/gemcitabine/vinorelbine (IGeV), 3.

[l] High-dose cytarabine, 1; high-dose cytarabine/mitoxantrone (HAM), 1.

[m] Cladribine/etoposide/cytarabine/G-CSF (CLEAG), 2; cladribine/vincristine/bortezomib (CLAOB), 1.

[n] Lenalidomide alone, 10; lenalidomide/rituximab, 2; lenalidomide/tafasitamab, 1; lenalidomide/trofosfamide/rituximab, 1; lenalidomide/methotrexate/leucovorin/cytarabine/rituximab (LeMLAR), 3.

[o] Brentuximab vedotin, 4; alemtuzumab, 1; crizotinib, 2; everolimus, 1; ibrutinib, 1.

[p] Induction therapy: GDP, 3; DexaBEAM, 2; DHAP/DexaBEAM, 3; DHAP/ICE, 2; German Burkitt's lymphoma protocol, 3; GemOx, 2; methotrexate/cytarabine/thiotepa-based protocols for central nervous system relapse, 4; other protocols, 7. High-dose therapy: BEAM, 17; thiotepa/etoposide/cytarabine/melphalan (TEAM), 1; thiotepa/carmustine/cyclophosphamide, 1; thiotepa/carmustine/etoposide, 1; thiotepa/carmustine, 1; thiotepa, 1; melphalan, 1; cyclophosphamide/total body irradiation, 1; unknown, 2. One patient received two lines of high-dose chemotherapy with autologous blood stem cell transplantation (for systemic and central nervous system relapse, respectively).

[q] Donor source, conditioning regimen, post-transplant immunosuppression, and donor lymphocyte infusion were not systematically recorded.

[r] Nivolumab, 1; nivolumab/urelumab, 1.

[s] In four cases combined with rituximab.

Supplemental Table S4

Logistic regression analysis of variables with potential impact on the risk of not proceeding to high-dose chemotherapy with autologous blood stem cell transplantation (autoSCT) in 94 autoSCT-eligible patients who were treated according to PETAL trial recommendations for first relapse.

Independent variable	Odds ratio	95% confidence interval	p[a]
Gender			
Male (reference)	1.000		
Female	1.724	0.665 – 4.467	0.262
Age			
≤ 60 years (reference)	1.000		
> 60 years	1.558	0.546 – 4.441	0.407
Type of lymphoma			
B cell lymphoma (reference)	1.000		
T cell lymphoma	2.253	0.634 – 8.005	0.209
Interval (continuous variable)			
Risk per month	0.962	0.931 – 0.994	0.019
Stage at relapse			
Stage I (reference)	1.000		
Stage II-IV[b]	2.267	0.408 – 12.615	0.350

[a] Wald test.

[b] Including 20 patients with unknown stage whose disease course was indistinguishable from that of stage IV patients (Figure 4).

At first relapse, all 94 patients were treated with at least one cycle of (R-)DHAP (median number of cycles, 2 [range, 1-6]), but 44 of these 94 patients (46.8%) were unable to proceed to high-dose chemotherapy with BEAM(-R) and autoSCT. Nagelkerke's $R^2 = 0.190$.

3. Supplemental Figures

Supplemental Figure S1

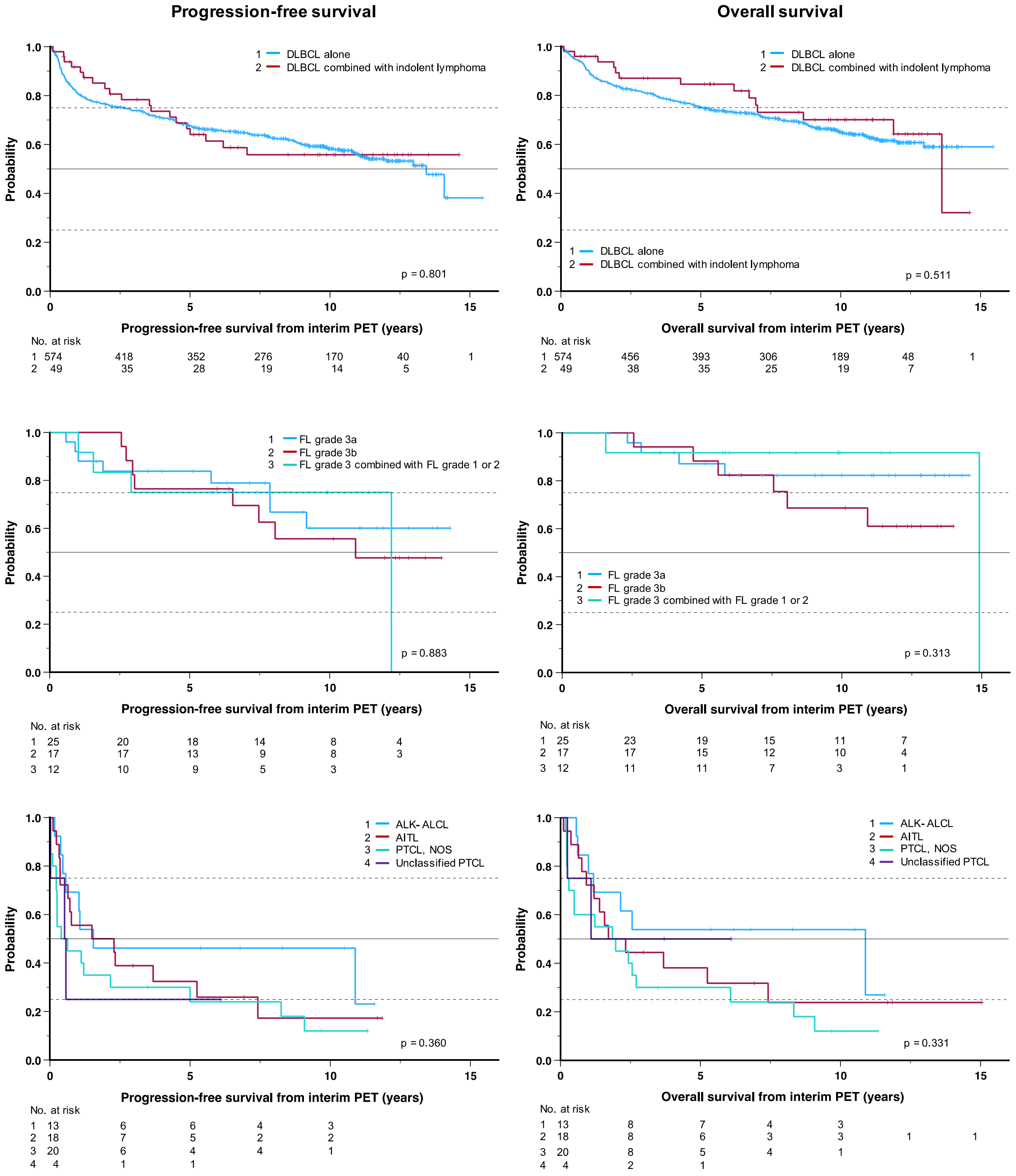

Progression-free survival (left) and overall survival (right) from the day of interim positron emission tomography (PET) for lymphoma subtypes that were combined in the present analysis.

Top, diffuse large B-cell lymphoma (DLBCL) alone versus DLBCL combined with an indolent lymphoma; middle, follicular lymphoma (FL) grade 3a versus FL grade 3b versus FL grade 3 combined with FL grade 1 or 2; bottom, anaplastic lymphoma kinase-negative (ALK-) anaplastic large cell lymphoma (ALCL) versus angioimmunoblastic T-cell lymphoma (AITL) versus peripheral T-cell lymphoma (PTCL), not otherwise specified (NOS) versus unclassified PTCL. Relapse rates: DLBCL versus DLBCL combined with an indolent lymphoma, 145/574 (25.3%) versus 13/49 (26.5%), p=0.845; FL grade 3a versus FL grade 3b versus FL grade 3 combined with FL grade 1 or 2, 8/25 (32.0%) versus 8/17 (47.1%) versus 4/12 (33.3%), p=0.584; ALK- ALCL versus AITL versus PTCL, NOS versus unclassified PTCL, 8/13 (61.5%) versus 14/18 (77.8%) versus 17/20 (85.0%) versus 3/4 (75.0%), p=0.487. No., number; p, log-rank test.

Supplemental Figure S2

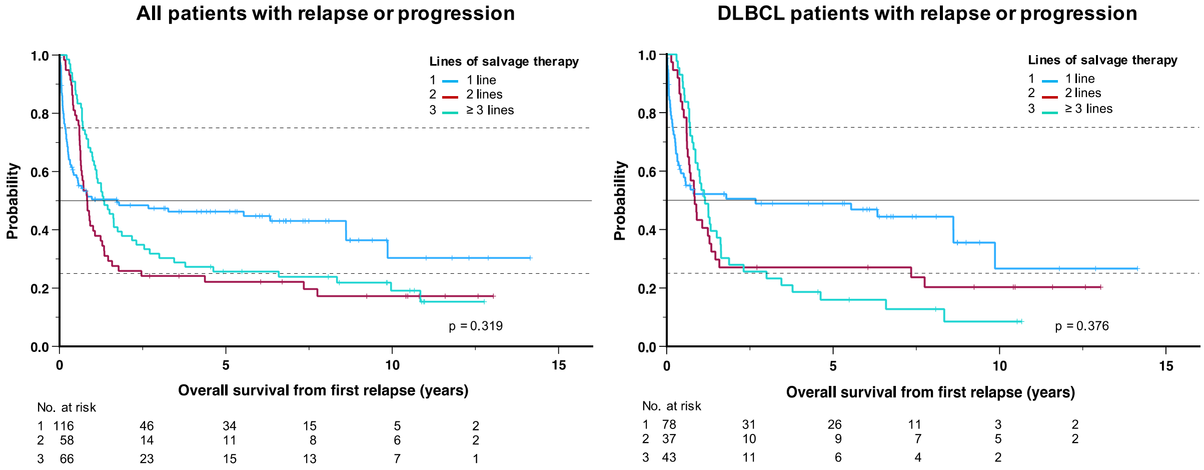

Overall survival from the day of first relapse in relation to the number of salvage therapy lines for all relapsing patients (left) and for the DLBCL subgroup (right).

Note that the survival curve for 1 line of salvage therapy (blue) crosses the curves for 2 (red) or ≥3 lines (green), indicating that the group of patients receiving only one line of salvage therapy is composed of two subgroups with different prognosis: About half of these patients are chemotherapy-refractory, succumbing to their disease within the first year after relapse, while the other half is chemotherapy-sensitive, deriving long-term benefit from salvage therapy. No., number; p, log-rank test.

Supplemental Figure S3

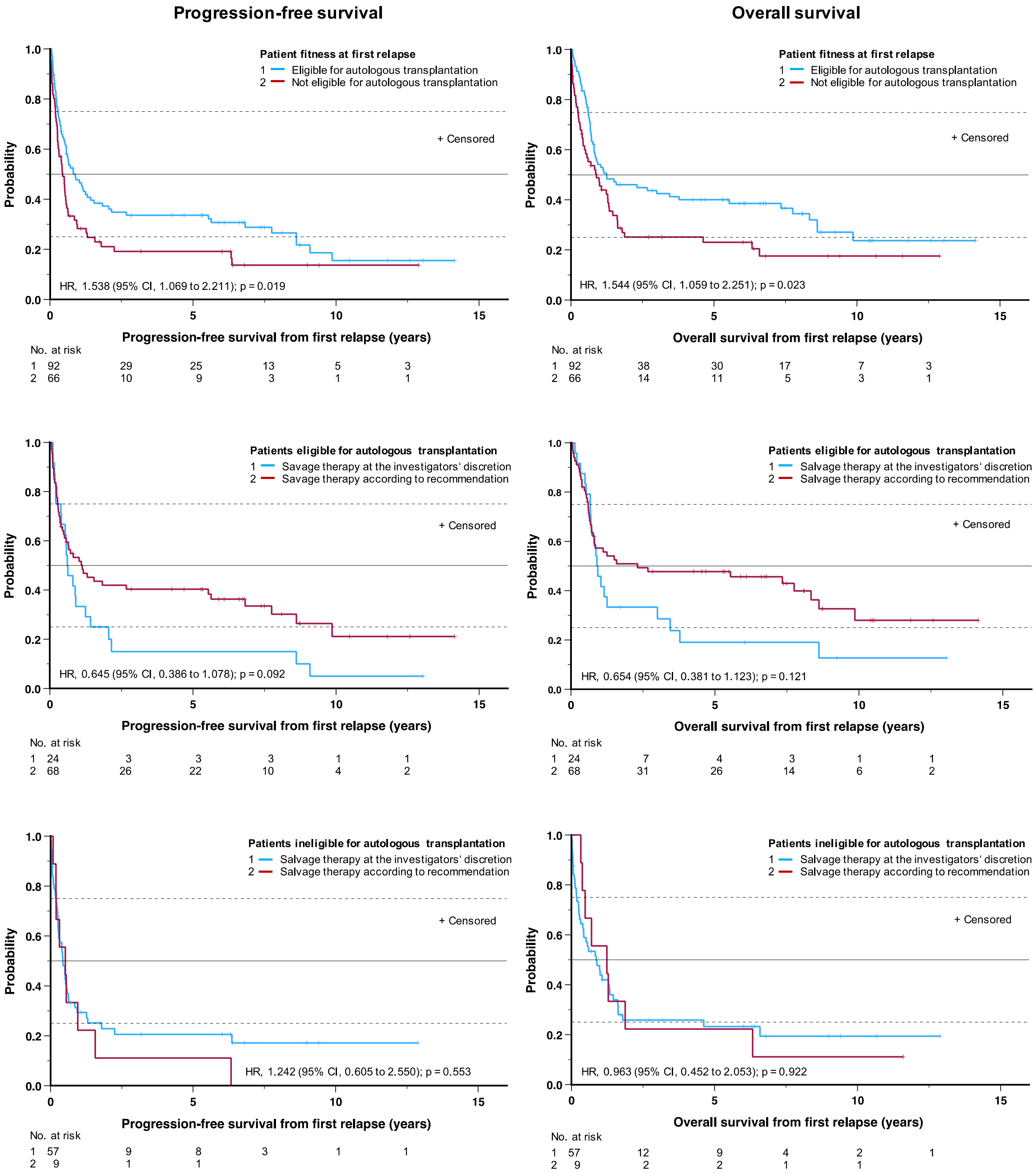

Progression-free survival (left) and overall survival (right) from the day of first relapse in the DLBCL subgroup in relation to eligibility for autologous blood stem cell transplantation (top), treatment of first relapse according to recommendation in transplant-eligible patients (middle), and treatment of first relapse according to recommendation in transplant-ineligible patients (bottom).
CI, confidence interval; HR, hazard ratio; No., number; p, log-rank test.

Supplemental Figure S4

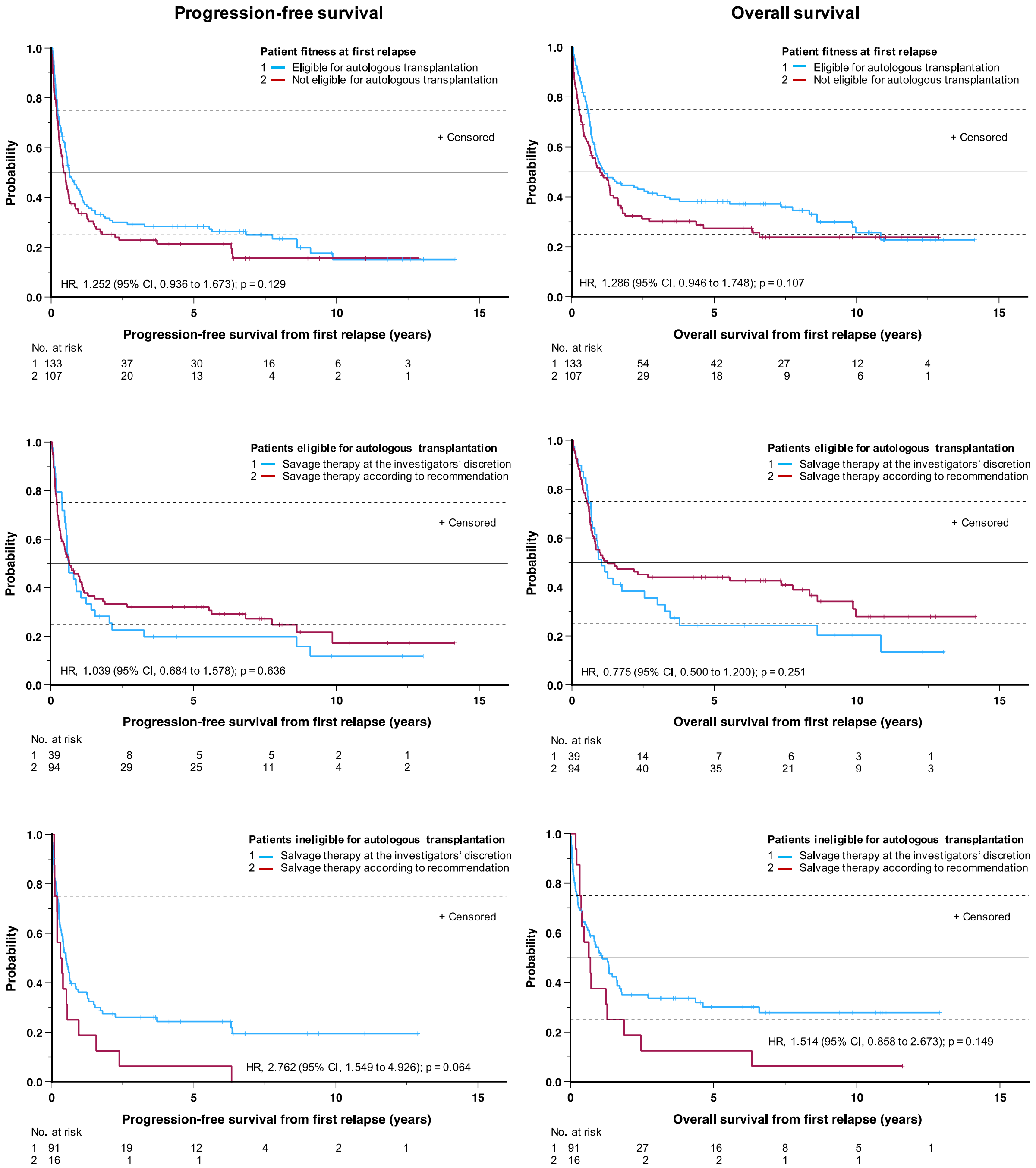

Progression-free survival (left) and overall survival (right) from the day of first relapse in the entire study population in relation to eligibility for autologous transplantation (top), treatment of first relapse according to recommendation in transplant-eligible patients (middle), and treatment of first relapse according to recommendation in transplant-ineligible patients (bottom).

Note that, in the bottom panels, seven patients deemed transplant-ineligible at first relapse were considered transplant-eligible in later lines of salvage therapy, but only one underwent autologous transplantation (treatment arm 2) and two underwent allogeneic transplantation (without preceding autologous transplantation, arm 1). CI, confidence interval; HR, hazard ratio; No., number; p, log-rank test.

Supplemental Figure S5

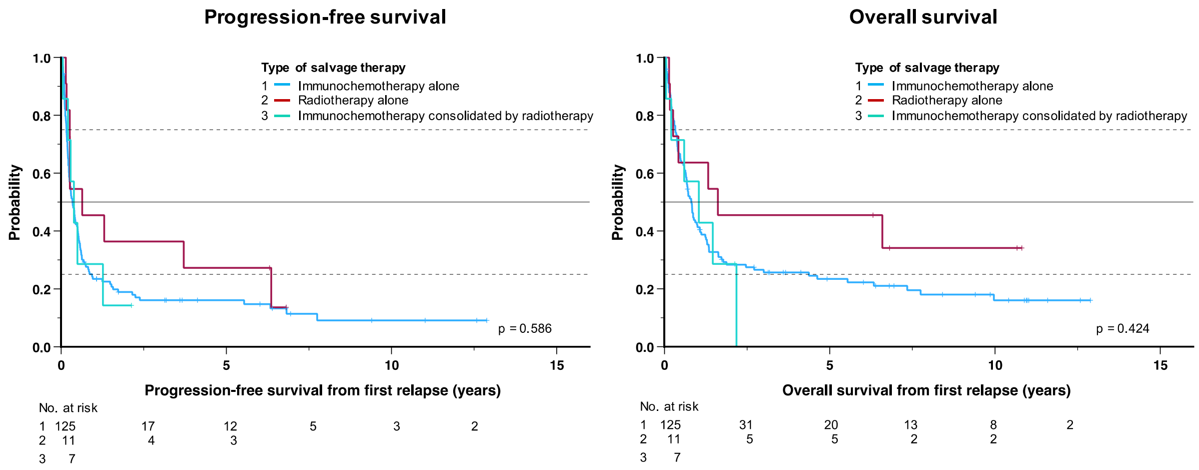

Progression-free survival (left) and overall survival (right) from the day of first relapse in relation to the type of salvage therapy applied in first relapse (excluding transplantation).

Stage I relapse was more common among patients treated with radiotherapy alone (3/11, 27.3%) than among patients treated with immunochemotherapy alone (9/125, 7.2%) or immunochemotherapy consolidated by radiotherapy (0/7, 0.0%; p=0.050). No., number; p, log-rank test.

4. Full list of sites, principal investigators, biostatisticians, reference pathologists, and members of the Data and Safety Monitoring Board

Sites and principal investigators

Oncological institutions (in alphabetical order of cities)

Aachen: Universitätsklinikum, S. Wilop, M. Tometten; **Berlin:** Charité Universitätsmedizin, A. Korfel, U. Keller; Vivantes Klinikum Neukölln, M. de Wit; **Bielefeld:** Evangelisches Krankenhaus, F. Weissinger; **Bocholt:** St. Agnes Krankenhaus, U. Stark; **Bochum:** Augusta-Kranken-Anstalt, D. Behringer; Gemeinschaftspraxis für Hämatologie, Onkologie, Hämostaseologie und Palliativmedizin, U. Bückner, H. Nückel; Knappschaftskrankenhaus, R. Schroers; **Bottrop:** Knappschaftskrankenhaus, G. Trenn; **Bremen:** Klinikum Bremen Mitte, B. Hertenstein; **Darmstadt:** Klinikum, H. Bernhard; **Dortmund:** Klinikum, M. Heike, M.-A. Wörns; **Dresden:** Universitätsklinikum Carl Gustav Carus, F. Kroschinsky; Onkologische Gemeinschaftspraxis, G. Prange-Krex; **Duisburg:** HELIOS St. Johannes Klinik, A. Giagounidis, G.U. Grigoleit; Medizinisches Versorgungszentrum Onkologie, Standort Duisburg-Nord, J. Selbach; Sana Kliniken, S. Petrasch, J.S. Balleisen; **Düsseldorf:** Marien Hospital, J. Schütte, A. Giagounidis; Universitätsklinikum, A. Dienst, U. Germing; **Essen:** Universitätsklinikum, U. Dührsen; Medizinisches Versorgungszentrum Hämatologie Onkologie, U. von Verschuer; Medizinisches Versorgungszentrum Essen-Nord, B. Schramm-Groß; Evangelisches Krankenhaus Werden, P. Reimer; **Fulda:** Klinikum, H.-G. Höffkes; **Goch:** Wilhelm-Anton-Hospital, V. Runde; **Halle/Saale:** Hämatologisch-onkologische Gemeinschaftspraxis, C. Spohn, R. Moeller; **Hamm:** St. Marien-Hospital, H. Dürk; **Hannover:** Medizinische Hochschule, D. Kofahl-Krause, M. Heuser; **Heidelberg:** Universitätsklinikum, M. Witzens-Harig, C. Müller-Tidow; **Heilbronn:** Klinikum am Gesundbrunnen, U.M. Martens; **Herne:** Marien Hospital, D. Strumberg; **Jena:** Universitätsklinikum, P. La Rosée, A. Hochhaus; **Kaiserslautern:** Westpfalz-Klinikum, H. Link, G. Held; **Kiel:** Universitätsklinikum Schleswig-Holstein, M. Kneba, C. Baldus; **Koblenz:** Stiftungsklinikum Mittelrhein, R. Naumann, J.M. Chemnitz; **Köln:** Krankenhaus Holweide, K. Schulte, C. Limmroth; **Leipzig:** Onkopraxis Probstheida, A. Schwarzer; Universitätsklinikum, D. Niederwieser, U. Platzbecker; **Lüdenscheid:** Klinikum, G. Heil, M. Schwalenberg; **Minden:** Johannes Wesling Klinikum, M. Grieshammer; **Mönchengladbach:** Medizinisches Versorgungszentrum Onkologie, C. Beck, M. Stephany; **Münster:** Universitätsklinikum, R. Mesters, I.E. Karsten; **Neumünster:** Friedrich-Ebert-Krankenhaus, H. Held, S. Mahlmann; **Oberhausen:** Praxis für Hämatologie und Onkologie, H. Steiniger; **Paderborn:** Brüderkrankenhaus St. Josef, T. Gaska; **Passau:** Klinikum, T. Südhoff; **Porta Westfalica:** Zentrum für Hämatologie und Onkologie Medizinisches Versorgungszentrum, C. Kreisel-Büstgens, E. Moorahrend; **Potsdam:** Klinikum Ernst von Bergmann, G. Maschmeyer, K. Jordan; **Recklinghausen:**

Knappschaftskrankenhaus, O. Kloke, M. Klein; Prosper-Hospital, T. Höhler; **Regensburg:** Universitätsklinikum, M. Grube, W. Herr; **Stuttgart:** Klinikum, D. Hahn; **Wuppertal:** HELIOS Universitätsklinikum, A. Raghavachar, O. Schmalz; Medizinisches Versorgungszentrum West, W. Fett; Petrus-Krankenhaus, M. Sandmann.

Nuclear medicine institutions (in alphabetical order of cities)

Aachen: Universitätsklinikum, T. Krohn; **Berlin:** Charité Universitätsmedizin, W. Brenner; Vivantes Klinikum Neukölln, M. Plotkin; **Bremen:** Zentrum für moderne Diagnostik (Zemodi), C. Franzius; **Dresden:** Universitätsklinikum Carl Gustav Carus, J. Kotzerke; **Düsseldorf/Jülich:** Universitätsklinikum, H. Hautzel; **Essen:** Universitätsklinikum, A. Bockisch; **Fulda:** Klinikum, A. Hertel; **Hannover:** Medizinische Hochschule, F. M. Bengel; **Heidelberg:** Universitätsklinikum, U. Haberkorn; **Jena:** Universitätsklinikum, M. Freesmeyer; **Kiel:** Universitätsklinikum Schleswig-Holstein, U. Lützen; **Koblenz:** Bundeswehrkranken-haus, A. Klein; **Leipzig:** Universitätsklinikum, R. Kluge; **Lüdenscheid:** Klinikum, R. Larisch; **Minden:** Johannes Wesling Klinikum, E. Fricke, J. Holzinger; **Mönchengladbach:** Kliniken Maria Hilf, W. Schäfer; **Münster:** Universitätsklinikum, M. Weckesser; **Paderborn:** Brüderkrankenhaus St. Josef, F. Nyuyki; **Passau:** Klinikum, W. Römer; **Potsdam:** Klinikum Ernst von Bergmann, I. Brink; **Regensburg:** Universitätsklinikum, J. Marienhagen; **Stuttgart:** Klinikum, G. Pöpperl.

Medical biometry and biostatistics

Essen: Institut für Medizinische Informatik, Biometrie und Epidemiologie, Universität Duisburg-Essen, K.-H. Jöckel, M. Nonnemacher, J. Rekowski, A. Scherag, M. Neuhäuser.

Reference pathologists (in alphabetical order)

A.C. Feller, Universitätsklinikum Schleswig-Holstein, Campus Lübeck

M.L. Hansmann, Universitätsklinikum, Frankfurt/Main

W. Klapper, Universitätsklinikum Schleswig-Holstein, Campus Kiel (chairman)

P. Möller, Universitätsklinikum, Ulm

A. Rosenwald, Universitätsklinikum, Würzburg

H. Stein, Universitätsklinikum Benjamin Franklin, Berlin

Data and Safety Monitoring Board (in alphabetical order)

R. Andreesen, Universitätsklinikum, Regensburg (oncology)

F. Grünwald, Universitätsklinikum, Frankfurt/Main (nuclear medicine)

D. Hoelzer, Onkologikum, Frankfurt/Main (oncology, chairman)

W. Köpcke, Universitätsklinikum, Münster (biostatistics)

Positron-emission tomography-guided therapy of aggressive non-Hodgkin lymphomas (PETAL trial)

English synopsis of the most recent version of the protocol

(provided by Ulrich Dührsen, MD, principal investigator)

<u>Protocol identification:</u>

Protocol code:	PETAL trial
EudraCT number:	2006-001641-33
ClinicalTrials.gov number:	NCT00554164
Protocol version:	2.02
Date of protocol version:	May 1st, 2011

Supported by Deutsche Krebshilfe

This English synopsis of version 2.02 of the trial protocol also contains information about previous protocol versions according to which the trial was conducted before the present version became effective.

1. Selection of patients

1.1 Eligibility criteria

Histological diagnosis of an aggressive non-Hodgkin lymphoma (1):

B-cell lymphomas

Diffuse large B-cell lymphoma including variants, subtypes and borderline cases

Primary mediastinal large B-cell lymphoma

Intravascular large B-cell lymphoma

Plasmablastic lymphoma

Follicular lymphoma grade 3a or grade 3b

T-cell lymphomas

ALK-positive anaplastic large cell lymphoma
ALK-negative anaplastic large cell lymphoma
Angioimmunoblastic T-cell lymphoma
Peripheral T-cell lymphoma, not otherwise specified
Hepatosplenic gamma-delta T-cell lymphoma
Subcutaneous panniculitis-like T-cell lymphoma

Baseline positron-emission tomography (PET) with pathological (^{18}F)-fluorodeoxy-glucose (FDG) uptake

Performance status ECOG 0 - 3

Age 18 - 80 years

Effective contraception

Ability to understand the goal of the study and act accordingly

Geographical proximity to a study center for treatment and follow-up

Compliance

Written informed consent

No ineligibility criteria

1.2 Ineligibility criteria

Burkitt's lymphoma, primary cerebral lymphoma, lymphoblastic lymphoma, transformed indolent lymphoma, non-leg-type primary cutaneous large cell lymphoma

Start of treatment before baseline FDG-PET

Previous chemo- and/or radiotherapy (except radioiodine therapy of the thyroid)

Other neoplastic diseases in the preceding 5 years (except in situ carcinoma or basalioma)

Active viral hepatitis (hepatitis B [HBV]: HbsAg positive or HBV DNA positive; hepatitis C [HCV]: anti-HCV positive), human immunodeficiency virus infection, other uncontrolled infections

Severe organ dysfunction (not explained by the lymphoma) precluding treatment according to protocol

Institutionalization by court order

Pregnancy or lactation

2. Schema and treatment plan

2.1 Overview

Patients with a positive baseline PET scan received two cycles (R-)CHOP followed by interim PET scanning. The use of rituximab was restricted to patients with CD20-positive lymphomas. Throughout the treatment plan, the (R-)CHOP protocol was delivered in 14-day intervals (R-CHOP14), except for the interval between the second and the third cycle which was 21 days (to avoid false-positive interim PET results). The interim PET result was expedited to the trial office for group assignment (positive versus negative findings) and randomization (1:1) which was done centrally by the Center for Clinical Studies of the University of Duisburg-Essen using the Pocock and Simon minimization method with dynamic allocation (2). Stratification variables were age (18-50, 51-60, 61-70, 71-80 years), sex, risk group of the International Prognostic Index (IPI) (3), CD20 status, and treatment center (able to deliver all parts of the protocol vs. able to deliver only (R-)CHOP, i.e. need to cooperate with another trial site for the delivery of the Burkitt protocol which required hospitalization).

Patients with a positive interim scan were randomly assigned to receive six additional two-weekly cycles (R-)CHOP or six three-weekly blocks of the Burkitt protocol. The treatment intensity of the Burkitt protocol was lower in patients aged 61 to 80 years than in patients aged 18 to 60 years (4).

From November 2007 through January 2010, patients with a negative interim PET scan were uniformly treated with four additional two-weekly cycles (R-)CHOP. When publications suggested that increasing the dose of rituximab may improve treatment results (5,6), the protocol was amended to test this hypothesis in patients with CD20-positive lymphomas and a negative interim PET scan. From February 2010 through October 2011, such patients were randomly assigned to receive four additional cycles R-CHOP or the same treatment with two extra two-weekly doses rituximab. When the recruitment goal was reached, randomization was stopped. From November 2011 to the end of recruitment in December 2012, patients with CD20-positive lymphomas and a negative interim PET scan uniformly received four additional cycles R-CHOP with two extra doses rituximab which, by then, was considered the standard of care for aggressive B-cell lymphomas in Germany. Patients with CD20-negative lymphomas and a negative interim PET scan did not undergo randomization at any time. After the interim scan, they uniformly received four additional two-weekly cycles CHOP.

The study protocol made specific recommendations for the treatment of progression or relapse (cisplatin-based salvage therapy with or without high-dose therapy with autologous blood stem cell transplantation, see section 2.3).

2.2 Flow chart

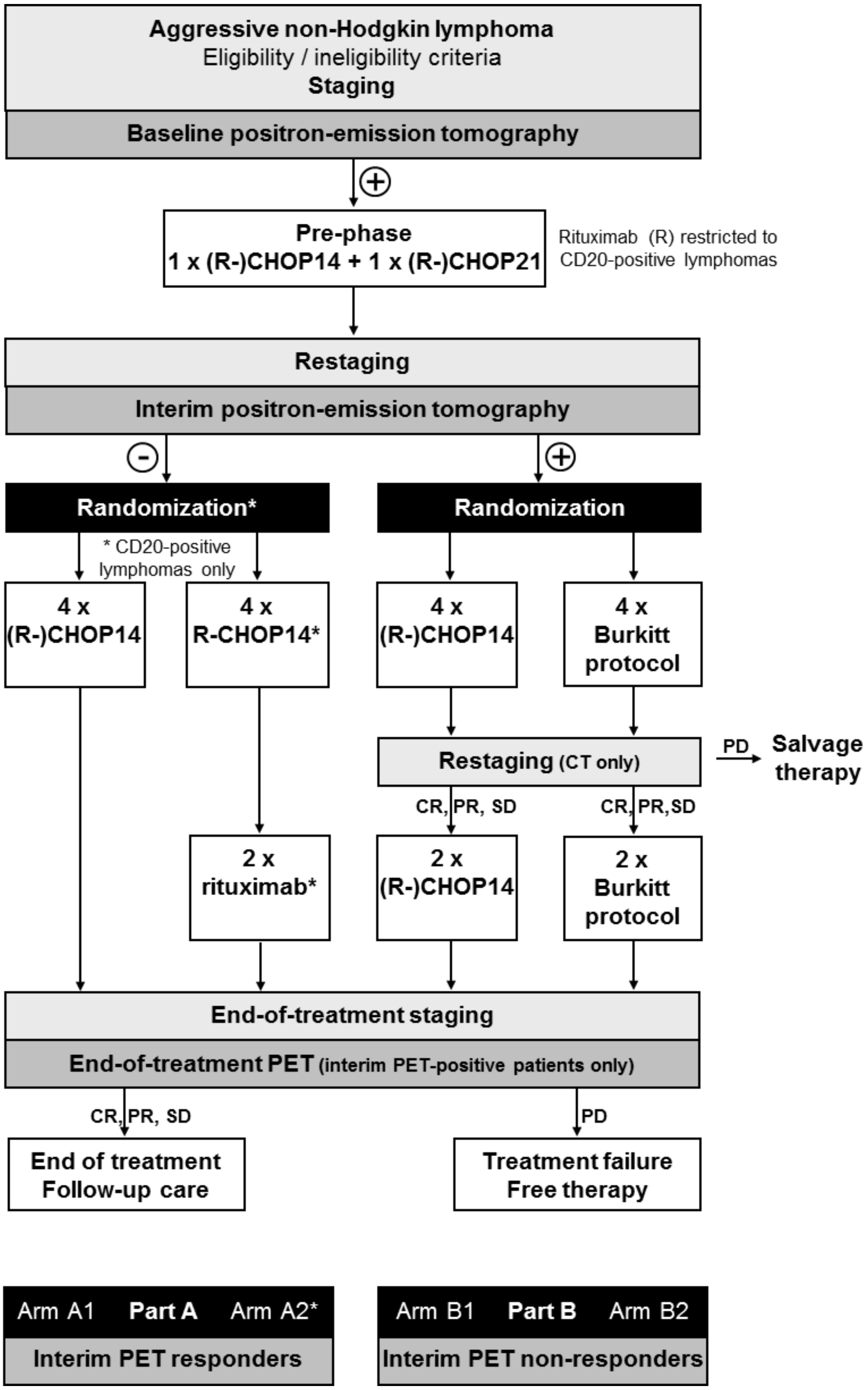

2.3 Administration schedule

Administration of cytotoxic agents or glucocorticoids before baseline PET scanning was not allowed. After the baseline investigations, all patients received a therapy pre-phase consisting of vincristine (1 mg, day 1) and prednisone (100 mg for 3 to 7 days) (5).

The (R-)CHOP regimen was delivered either on an outpatient or on an inpatient basis. The Burkitt protocol required hospitalization for a minimum of 6 days.

In both protocols, the use of rituximab was restricted to CD20-positive lymphomas. Cyclophosphamide and ifosfamide were given with MESNA uroprotection. All treatment cycles except cycle 2 were given with granulocyte colony-stimulating factor support which was started on day 5 or day 7 of the (R-)CHOP or Burkitt protocols, respectively, and continued until recovery of neutrophils >1/nl. In case of toxicity, dose reductions or treatment delays were made as outlined below. Patients with a high risk of central nervous system relapse (≥4 risk factors as defined by Hollender et al [7]) received a total of 4 intrathecal methotrexate injections (15 mg each with oral leucovorin rescue) on day 3 of the first 4 therapy cycles. Infection prophylaxis (e.g. oral cotrimoxazole, colistin and amphotericin B) was mandatory during treatment with the Burkitt protocol and optional during treatment with (R-) CHOP. Abbreviations: iv, intravenous; po, per os; hrs, hours.

R-CHOP

Rituximab	375 mg/m² iv	day 1
Cyclophosphamide	750 mg/m² iv	day 2
Doxorubicin	50 mg/m² iv	day 2
Vincristine	2 mg iv	day 2
Prednisone	100 mg po	days 2 to 6

Cycle intervals were 14 days, except the interval between cycles 2 and 3 which was 21 days.

Burkitt protocol

The Burkitt protocol consisted of three 5-day chemotherapy blocks termed A, B and C, of which patients below the age of 60 years received the sequence A – B – C – A – B – C, while patients between 60 and 80 years received a dose-reduced version of the sequence A – B – A – B – A – B. To avoid undue myelotoxicity block C containing high-doses of cytarabine and etoposide was not given to patients above the age of 60 years (4).

Methotrexate was given as a continuous 24-hour infusion followed by leucovorin rescue. Cytarabine was given twice daily with a 12-hour interval between infusions. High-dose cytarabine was given as a 3-hour infusion. Cycle intervals were 21 days.

Patients ≤ 60 years

Block A (≤ 60 years)

Rituximab	375 mg/m² iv	day 1
Dexamethasone	10 mg/m² po	days 2 to 6
Vincristine	2 mg iv	day 2
Methotrexate	1500 mg/m² (iv 24 hrs)	day 2
Patients > 55 years:	500 mg/m² (iv 24 hrs)	
Ifosfamide	800 mg/m² iv	days 2 to 6
Cytarabine	2 x 150 mg/m² iv	days 5 and 6
Etoposide	100 mg/m² iv	days 5 and 6

Block B (≤ 60 years)

Rituximab	375 mg/m² iv	day 1
Dexamethasone	10 mg/m² po	day 2 to 6
Vincristine	2 mg iv	day 2
Methotrexate	1500 mg/m² (iv 24 hrs)	day 2
Patients > 55 years:	500 mg/m² (iv 24 hrs)	
Cyclophosphamide	200 mg/m² iv	days 2 to 6
Doxorubicin	25 mg/m² iv	days 5 and 6

Block C (≤ 60 years)

Rituximab	375 mg/m² iv	day 1
Dexamethasone	10 mg/m² po	days 2 to 6
Vindesine	3 mg/m² iv (max. 5 mg)	day 2
Methotrexate	1500 mg/m² (iv 24 hrs)	day 2
Patients > 55 years:	500 mg/m² (iv 24 hrs)	
Etoposide	250 mg/m² iv	days 5 and 6
Cytarabine	2 x 2.000 mg/m² (iv 3 hrs)	day 6
Patients > 55 years:	2 x 1.000 mg/m² (iv 3 hrs)	

Patients > 60 years

Block A (> 60 years)

Rituximab	375 mg/m² iv	day 1
Dexamethasone	10 mg/m² po	days 2 to 6
Methotrexate	500 mg/m² (iv 24 hrs)	day 2
Ifosfamide	400 mg/m² iv	days 2 to 6
Cytarabine	2 x 60 mg/m² iv	days 5 and 6
Etoposide	60 mg/m² iv	days 5 and 6

Block B (> 60 years)

Rituximab	375 mg/m² iv	day 1
Dexamethasone	10 mg/m² po	days 2 to 6
Vincristine	1 mg iv	day 2
Methotrexate	500 mg/m² (iv 24 hrs)	day 2
Cyclophosphamide	200 mg/m² iv	days 2 to 6
Doxorubicin	25 mg/m² iv	days 5 and 6

Salvage regimens for patients with refractory disease or relapse

The trial protocol also contained treatment recommendations for patients progressing on therapy or relapsing after the end of therapy. Patients below the age of 60 years were to receive two cycles of (R-)DHAP (dexamethasone, high-dose cytarabine, cisplatin) followed by high-dose (R-)BEAM (carmustine, etoposide, cytarabine, melphalan) with autologous blood stem cell transplantation (8), while patients between 60 and 80 years were to receive six cycles of the (R-)ESHAP protocol (etoposide, methylprednisolone, high-dose cytarabine, cisplatin) (9).

Since the protocol did not specify whether the age limit pertained to the age at study inclusion or the age at relapse and personal experience and published reports (10,11) indicated that high-dose chemotherapy with autologous blood stem cell transplantation can be safely administered to fit patients above age 60, while unfit patients below age 60 might not be candidates for the procedure, the recommendations were used for patients deemed eligible or ineligible for transplantation irrespective of age. Eligibility for autologous blood stem cell transplantation was defined by the treating physicians without involvement of the trial office.

Patients eligible for high-dose therapy and autologous blood stem cell transplantation

Two cycles of (R-)DHAP (3-week interval)

Rituximab	375 mg/m² iv	day 1
Dexamethasone	40 mg po	days 2 to 5
Cytarabine	2 x 2.000 mg/m² 3h CI	day 3
Cisplatin	100 mg/m² 24h CI	day 2
G-CSF	5 µg/kg daily sc<br>or<br>6 mg single-shot sc	starting on day 4 until recovery of neutrophils or<br>depot preparation given on day 4

In patients with a positive interim PET scan, peripheral blood stem cells were collected immediately after the first treatment cycle following the interim PET evaluation (i.e., after either (R-)CHOP or block A of the Burkitt protocol) and stored until needed. In patients relapsing despite a negative interim scan, the stem cells were harvested after the second cycle of (R-)DHAP. Rituximab was restricted to patients with CD20-positive lymphomas. Computed tomography-based restaging was performed after the second cycle. In case of complete or partial remission, the patients proceeded to high-dose chemotherapy with BEAM(-R). CI, continuous infusion; h, hour.

One cycle of BEAM(-R) followed by autologous blood stem cell transplantation

Carmustine	300 mg/m² iv	day -6
Etoposide	200 mg/m² iv	days -6 to -3
Cytarabine	2 x 200 mg/m² iv	days -6 to -3
Melphalan	140 mg/m² iv	day -2
Blood stem cells	≥ 2 x 10^6 CD34+ cells/kg iv	day 0
Rituximab	375 mg/m² iv	days +1 and +8

Rituximab was restricted to CD20-positive lymphomas.

Patients ineligible for high-dose therapy and autologous blood stem cell transplantation

Up to 6 cycles of (R-)ESHAP (3-week intervals)

Rituximab	375 mg/m² iv	day 1
Etoposide	40 mg/m² iv	days 2 to 5
Methylprednisolone	500 mg iv	days 2 to 6
Cytarabine	2.000 mg/m² 3h CI	day 6
Cisplatin	25 mg/m² 24h CI	days 2 to 5
G-CSF	5 µg/kg daily sc<br>or<br>6 mg single-shot sc	starting on day 7 until recovery of neutrophils or<br>depot preparation given on day 7

Rituximab was restricted to patients with CD20-positive lymphomas. Computed tomography-based restaging was performed after the third cycle. In case of complete or partial remission, the patients received another 3 cycles of (R-)ESHAP. CI, continuous infusion; h, hour.

3. Rules for dose modification

3.1 Treatment delays

A treatment cycle was given as planned if the following conditions were met:

- hematopoietic recovery beyond leukocyte and platelet nadir
- leukocytes >2.5/nl or neutrophils >1.0/nl
- platelets >80/nl
- no active infection
- no other serious toxicity or organ dysfunction

If the conditions were not fulfilled on the planned day of treatment, the cycle was delayed by 3 days. If after that time the conditions still failed to be fulfilled, the cycle was delayed by another 3 - 4 days. If the delay exceeded one week, the doses of the major myelotoxic agents were reduced in subsequent treatment cycles according to the following scheme:

<u>Dose reduction in case of treatment delay by 1 - 2 weeks</u>

<u>R-CHOP</u>

Cyclophosphamide	75% of planned dose
Doxorubicin	75% of planned dose

<u>Burkitt protocol</u>

Ifosfamide	75% of planned dose
Cyclophosphamide	75% of planned dose
Doxorubicin	75% of planned dose
Etoposide	75% of planned dose
Cytarabine	50% of planned dose

<u>R-DHAP</u>

Cisplatin	75 % of planned dose
Cytarabine	50 % of planned dose

<u>R-ESHAP</u>

Etoposide	75 % of planned dose
Cisplatin	75 % of planned dose<br>(3 instead of 4 days of continuous infusion)
Cytarabine	50 % of planned dose

Dose reduction in case of treatment delay by >2 weeks

R-CHOP

Cyclophosphamide	50% of planned dose
Doxorubicin	50% of planned dose

Burkitt protocol

Ifosfamide	50% of planned dose
Cyclophosphamide	50% of planned dose
Doxorubicin	50% of planned dose
Etoposide	50% of planned dose
Cytarabine	25% of planned dose

R-DHAP

Cisplatin	50 % of planned dose
Cytarabine	25 % of planned dose

R-ESHAP

Etoposide	50 % of planned dose
Cisplatin	50 % of planned dose<br>(2 instead of 4 days of continuous infusion)
Cytarabine	25 % of planned dose

3.2 Dose reductions

Impaired renal function

Ifosfamide, cisplatin, melphalan

Creatinine <2 mg/dl	full dose
Creatinine 2 - 3 mg/dl	50% of planned dose
Creatinine >3 mg/dl	no ifosfamide, cisplatin or melphalan

Methotrexate

The methotrexate dose was adjusted according to the creatinine clearance as determined by the Cockcroft-Gault formula (http://www.nephron.com/cgi-bin/CGSI.cgi).

Creatinine clearance	≥100 ml/min	100% of planned dose
	50 - 99 ml	creatinine clearance (ml/min) = % of planned dose to be administered)<br>i.e., clearance 75 ml/min → 75% of planned methotrexate dose
	<50 ml/min	10% of planned dose or omission of methotrexate

Impaired hepatic function

Doxorubicin, vincristine, vindesine

Bilirubin <2 mg/dl	full dose
Bilirubin 2 - 3 mg/dl	50% of planned dose
Bilirubin >3 mg/dl	no doxorubicin, vincristine or vindesine

Neurotoxicity

Vincristine, vindesine

≥grade 2 sensory neuropathy	50% of planned dose
≥grade 2 motor neuropathy or ileus	no vincristine or vindesine

Ifosfamide

Psychosis	replacement of ifosfamide by cyclophosphamide (dose [mg] = 20% of ifosfamide dose [mg])

Ototoxicity

Cisplatin

Moderate hearing loss	75% of planned dose
Severe hearing loss	50% of planned dose
Impending deafness	no cisplatin

Mucositis

Methotrexate

In case of severe mucositis, co-administration of drugs interfering with methotrexate binding or elimination (e.g. salicylic acid, non-steroidal anti-rheumatic drugs, penicillin, sulfonamides, vitamin C) and accumulation in a third space (e.g., ascites, pleural effusion) had to be excluded.

Repeated grade 4 mucositis	Shortening of infusion time from 24 to 12 hrs; if no improvement, shortening of infusion time to 6 hrs

If shortening of the infusion time led to a marked decrease in the severity of mucositis, the infusion time could gradually be increased again.

4. Measurement of treatment effect

4.1 Response criteria

Response was assessed using a modification of the 2007 revised response criteria for malignant lymphoma (12). Patients with a positive interim PET scan not progressing during the final 6 treatment cycles (as assessed by computed tomography after post-interim PET cycle 4, cf. flow chart) underwent end-of-treatment PET/CT scanning which was evaluated using the International Harmonization Project criteria (12). In patients with a negative interim PET scan, an end-of-treatment PET scan was not mandatory. In these patients, response was assessed by computed tomography as outlined in the 2007 revised response criteria for malignant lymphoma (12).

4.2 Survival

In patients undergoing randomization, event-free survival was measured from the day of randomization to disease progression, relapse, change of therapy incompatible with the trial protocol, toxicity-related discontinuation, or death from any cause, or censored at the date of last information. Progression-free survival was measured from the day of randomization to disease progression, relapse or death from any cause, and overall survival was measured from the day of randomization to death from any cause. In patients not undergoing randomization, survival was measured from the day of interim PET scanning. Interim PET scanning and randomization were done on the same day.

4.3 Methods of measurement

All assessments were done by the local investigators. After the end of recruitment, a random sample of 10% of all baseline and interim scans was reevaluated in a blinded fashion by independent investigators from the 10 most active nuclear medicine institutions with regard to classifying the interim scan as positive or negative, respectively. To this end the original PET or PET/CT images as reconstructed by the scanning centers were sent to the reference centers and read without clinical information using the local workstations and software. Reevaluation of baseline and interim PET scans by independent investigators was not part of the trial protocol, but a decision made by the nuclear medicine reference panel at an investigator meeting on May 20th, 2011.

<u>Baseline PET scanning</u>

A positive baseline scan required FDG uptake above background in at least one anatomical site (12). Subsequent investigations were done under the same conditions and on the same scanner as baseline scanning.

<u>Interim PET scanning</u>

Interim scanning was performed after the second (R-)CHOP cycle, with a chemotherapy-free interval of at least 10, preferably 20 days. Administration of granulocyte colony-stimulating factor (G-GSF) was not allowed after the second treatment cycle. Interim PET scans were evaluated semi-quantitatively by comparing the maximum standardized uptake value (SUV_{max}) within the manifestations of the lymphoma at interim PET with the SUV_{max} at baseline, irrespective of whether the SUV_{max} at baseline and the SUV_{max} at interim scanning were in the same or in different locations. Following the original description of the ΔSUV_{max} method (13), a positive interim PET was defined as a scan in which the SUV_{max} was reduced by ≤66% of the baseline SUV_{max}. Interim scans demonstrating an SUV_{max} reduction >66% were considered negative. In patients with low baseline SUV_{max} (<10), interim PET scans without unphysiological FDG uptake, as judged by visual criteria, were regarded as negative, even if the percentage reduction was ≤66%. This modification of the ΔSUV_{max} method allowed for the fact that, in patients with low baseline SUV_{max}, a return to physiological activity may require less than a 66% reduction.

<u>End-of-treatment PET scanning</u>

End-of-treatment PET scans were evaluated using the International Harmonization Project criteria whereby any remaining FDG activity above that of the mediastinal blood pool is rated as a pathological finding (12).

5. Reasons for early cessation of trial therapy

5.1 Early cessation in individual patients

- Progressive disease during treatment
- Change to a treatment regimen not described in the study protocol
- Intolerable toxicity
- Severe protocol violation
- Non-compliance
- Patient preference
- Decision made by the treating physician

5.2 Premature termination of the trial

The trial could be terminated prematurely,

- if the risks associated with continuation outweighed the expected benefits,
- if new scientific data cast doubt on the appropriateness of the trial,
- if recruitment was insufficient to reach the trial goals, or
- if there was an intolerably high number of protocol violations.

All trial subjects still under treatment at the time of termination had to undergo a final examination. The decision to terminate the trial had to be made by the protocol committee. If an agreement could not be reached, the protocol committee had to seek a recommendation from the Data and Safety Monitoring Board.

In 2011 a publication confirmed the experience made in the PETAL trial that, using the ΔSUV_{max} method for interim PET evaluation, the proportion of interim PET-positive patients was considerably lower than 50% (14). Because patient accrual to part B of the trial was slow and recruitment in part A was nearing completion, the protocol committee asked the principal investigator’s ethics committee for advice how to proceed. The ethics committee’s recommendation is the basis for version 2.02, May 1st 2011, of the trial protocol:

- continue recruitment in parts A and B of the protocol
- in December 2012, reveal all efficacy and safety data of part B to the independent Data and Safety Monitoring Board and seek advice with regard to continuation or premature termination of the trial
- stop recruitment in part A as soon as the recruitment goal is reached, and treat any additional patients with CD20-positive lymphomas and a negative interim PET scan uniformly according to arm A2 until recruitment in part B is terminated

6. Objectives and entire statistical section

6.1 Objectives

The PETAL trial was a multicenter, prospective, randomized, parallel group, controlled study designed to determine whether an early change from (R-)CHOP to an intensive Burkitt's lymphoma protocol could improve the event-free survival in patients with aggressive B-cell or T-cell lymphomas whose interim PET scan remained positive after the first two treatment cycles. In patients with CD20-positive lymphomas and a negative interim PET scan, the trial tested whether the addition of two doses rituximab to six cycles R-CHOP could improve the event-free survival. All primary and secondary endpoints were analyzed in the intention-to-treat population. The primary endpoint and a selection of secondary endpoints were also analyzed in the per-protocol population.

6.2 Endpoints

Primary endpoint (for first-line randomization)

Event-free survival

Secondary endpoints (for first-line and salvage therapy)

Post-treatment remission status
Time to progression
Progression-free survival
Overall survival
Proportion of patients with a positive or negative interim PET scan
Predictive value of interim PET scanning
Toxicity (from the first day to 30 days after the last day of treatment)
Adherence to recommendations for salvage therapy
Second primary malignancies
Cause of death

Planned subgroup analyses

Sex (male, female)
Age (4 groups: 18-50, 51-60, 61-70, 71-80 years; or 2 groups: 18-60, 61-80 years)
International Prognostic Index (IPI): low, low-intermediate, high-intermediate, high risk
Lymphoma subtype (diffuse large B-cell lymphoma, primary mediastinal large B-cell lymphoma, follicular lymphoma grade 3, other aggressive B-cell lymphomas, peripheral T-cell lymphomas and subtypes of these entities)
Treatment facility (able to deliver all parts of the protocol vs. able to deliver only [R-]CHOP)

Unplanned subgroup analysis

Lymphoma subtype (diffuse large B-cell lymphoma) by IPI risk group
Relapse by interval from interim PET, stage, treatment type, and line of therapy
Relapse by adherence to recommendations for salvage therapy
Relapse by eligibility for high-dose chemotherapy and autologous transplantation

Secondary endpoints and planned subgroup analyses which are not the subject of the manuscript, are not included in the list.

6.3 Sample size calculation

The initial sample size calculation aimed at the comparison of 6 x (R-)CHOP and the Burkitt protocol in part B of the trial (patients with a positive interim PET scan). Specifically, corresponding to a hazard ratio of 1.51 with a significance level of 5% for a two-sided log-rank test and a statistical power of 80%, the 2-year event-free survival rate was assumed to increase from 30% under the (R-)CHOP protocol to 45% under the Burkitt protocol (4,15). This resulted in a required sample size of 157 patients per treatment arm. With an expected drop-out of 10% the required sample size was increased to 174 per treatment arm. At this point in time, the ratio of patients with positive and negative interim PET scans was expected to be approximately 1:1. Thus, the number of patients in part A (observational study with a single treatment arm 4 x [R-]CHOP) was planned to be equal to the number of patients in part B resulting in a total sample size of 2 x 2 x 174 = 696 patients for the entire trial.

With the second amendment of the study protocol (October 1st, 2009), event-free survival was introduced as a primary endpoint for part A, now comparing the randomized treatment arms 4 x R-CHOP and 4 x R-CHOP + 2 x R in patients with CD20-positive lymphomas. Sample size calculation was then also conducted for part A. Similarly using a two-sided log-rank test with a significance level of 5% and a statistical power of 80%, the 2-year event-free survival rate was assumed to increase from 80% in the 4 x R-CHOP arm to 90% in the 4 x R-CHOP + 2 x R arm, corresponding to a hazard ratio of 2.12 (5,16,17). This yielded a required sample size of 115 patients per treatment arm which was adjusted for a 10% drop-out to result in a required sample of 128 patients per treatment arm.

6.4 Summary of the statistical analyses

The primary endpoint event-free survival was planned to be analyzed in the intention-to-treat population using a two-sided log-rank test with a significance level of 5% in both parts of the study. Planned sensitivity analyses for event-free survival included among others replacing the intention-to-treat with the per-protocol population as well as using the Cox model adjusting for the respective stratification factors of the randomization. Exploratory secondary analyses included time-to-event endpoints like time to progression, progression-free survival and overall survival that were also planned to be analyzed using the log-rank test. Remission rates and other secondary endpoints that can be expressed as rates were planned to be analyzed using Monte Carlo estimation for exact p-values of a chi-squared test. This was specified in the statistical analysis plan and is a deviation from the original study protocol, but this decision was made by the responsible biometrician before database closure due to the high computational resources needed for the originally planned Fisher's exact test. Toxicity was planned to be analyzed CTCAE-based and tested using a chi-squared test as described above.

7. References

1. Swerdlow SH, Campo E, Harris NL, et al (eds): WHO classification of tumours of the haematopoetic and lymphoid tissues. Lyon, France, IARC Press, 2008

2. Pocock SJ, Simon R. Sequential treatment assignment with balancing for prognostic factors in the controlled clinical trial. Biometrics 31:103-115 1975

3. A predictive model for aggressive non-Hodgkin's lymphoma. The International Non-Hodgkin's Lymphoma Prognostic Factors Project. N Engl J Med 329:987-994, 199

4. Hoelzer D, Walewski J, Döhner H, et al; German Multicenter Study Group for Adult Acute Lymphoblastic Leukemia. Improved outcome of adult Burkitt lymphoma/leukemia with rituximab and chemotherapy: report of a large prospective multicenter trial. Blood 124:3870-3879, 2014

5. Pfreundschuh M, Schubert J, Ziepert M, et al; German High-Grade Non-Hodgkin Lymphoma Study Group (DSHNHL). Six versus eight cycles of bi-weekly CHOP-14 with or without rituximab in elderly patients with aggressive CD20+ B-cell lymphomas: a randomised controlled trial (RICOVER-60). Lancet Oncol 9:105-116, 2008

6. Müller C, Murawski N, Wiesen MH, et al. The role of sex and weight on rituximab clearance and serum elimination half-life in elderly patients with DLBCL. Blood 119:3276-3284, 2012

7. Hollender A, Kvaloy S, Nome O, et al. Central nervous system involvement following diagnosis of non-Hodgkin's lymphoma: a risk model. Ann Oncol 13:1099-1107, 2002

8. Gisselbrecht C, Glass B, Mounier N, et al. Salvage regimens with autologous transplantation for relapsed large B-cell lymphoma in the rituximab era. J Clin Oncol 28:4184-4190, 2010

9. Velasquez WS, McLaughlin P, Tucker S, et al. ESHAP - an effective chemotherapy regimen in refractory and relapsing lymphoma: a 4-year follow-up study. J Clin Oncol 12:1169-1176, 1994

10. Moskowitz CH, Bertino JR, Glassman JR, et al. Ifosfamide, carboplatin, and etoposide: a highly effective cytoreduction and peripheral-blood progenitor-cell mobilization regimen for transplant-eligible patients with non-Hodgkin's lymphoma. J Clin Oncol 17:3776-3785, 1999

11. Caballero MD, Pérez-Simón JA, Iriondo A, et al. High-dose therapy in diffuse large cell lymphoma: results and prognostic factors in 452 patients from the GEL-TAMO Spanish Cooperative Group. Ann Oncol 14:140-151, 2003

12. Cheson BD, Pfistner B, Juweid ME, et al; International Harmonization Project on Lymphoma. Revised response criteria for malignant lymphoma. J Clin Oncol 25:579-586, 2007

13. Lin C, Itti E, Haioun C, et al. Early 18F-FDG PET for prediction of prognosis in patients with diffuse large B-cell lymphoma: SUV-based assessment versus visual analysis. J Nucl Med 48:1626-1632, 2007

14. Casasnovas RO, Meignan M, Berriolo-Riedinger A, et al; Groupe d'étude des lymphomes de l'adulte (GELA). SUVmax reduction improves early prognosis value of interim positron emission tomography scans in diffuse large B-cell lymphoma. Blood 118:37-43, 2011

15. Mikhaeel NG, Hutchings M, Fields PA, et al. FDG-PET after two to three cycles of chemotherapy predicts progression-free and overall survival in high-grade non-Hodgkin lymphoma. Ann Oncol 16:1514-1523, 2005

16. Brusamolino E, Rusconi C, Montalbetti L, et al. Dose-dense R-CHOP-14 supported by pegfilgrastim in patients with diffuse large B-cell lymphoma: a phase II study of feasibility and toxicity. Haematologica 91:496-502, 2006

17. Adde M, Enblad G, Hagberg H, et al. Outcome for young high-risk aggressive B-cell lymphoma patients treated with CHOEP-14 and rituximab (R-CHOEP-14). Med Oncol 23:283-293, 2006
